# Systematic Review of Loneliness and Social Isolation Interventions in Obesity and Obesity‐Related Complications

**DOI:** 10.1111/obr.70099

**Published:** 2026-01-26

**Authors:** Ghada Alsultany, Milan Piya, Kathryn Williams, Kate A. McBride

**Affiliations:** ^1^ Translational Health Research Institute Western Sydney University Penrith New South Wales Australia; ^2^ South Western Sydney (SWS) Metabolic Rehabilitation and Bariatric Program Camden and Campbelltown Hospitals Camden New South Wales Australia; ^3^ School of Medicine Western Sydney University Penrith New South Wales Australia; ^4^ Charles Perkins Centre‐Nepean and the Faculty of Medicine and Health The University of Sydney New South Wales Australia; ^5^ Department of Endocrinology Nepean Hospital Kingswood New South Wales Australia

**Keywords:** complications, loneliness, obesity, social isolation

## Abstract

People living with obesity and obesity‐related complications who are experiencing social isolation and loneliness (SIL) are at an increased risk of more disease‐specific complications, the presence of comorbidities, and mortality. Interventions targeting SIL may be of benefit in this population, though research in this field is limited. This study aimed to systematically review the literature on interventions addressing SIL in people with obesity and obesity‐related complications. The databases SCOPUS, PsycINFO, and EMBASE were searched for eligible articles. Studies were uploaded into Covidence for title, abstract, and full‐text screening, data extraction, and quality assessment. Of 3521 studies screened, 19 were included. Studies were grouped by whether they were conducted in person or through technology, and in group or individual settings, with in‐person group‐based interventions more likely to report effectiveness. Interventions were also divided into four types—therapeutic, companionship, social activity, or physical type—with studies included being predominantly therapeutic interventions. Limited conclusions could be drawn from the data in relation to effectiveness due to the heterogeneity of studies. Although the limited findings align with the emerging nature of this topic, it emphasizes the need for more research in developing targeted and robust SIL interventions for individuals with obesity and obesity‐related complications.

## 1 Introduction

Social isolation and/or loneliness (SIL) are social determinants of health and are important public health issues due to their significant impact on physical and mental health outcomes [1, 2]. Although often used interchangeably in the literature, loneliness and social isolation have distinct, albeit related, definitions. Loneliness is defined as the subjective perception of lack of close social ties [1, 3, 4], whereas social isolation is the objective quantification of diminished social ties. This may include limited social interactions, social networks, or support systems [1, 3, 4]. Although social isolation can lead to loneliness, socially isolated individuals may not necessarily feel lonely [1, 3]. Chronic SIL has been linked to cognitive decline, depression, cardiovascular disease, and increased risk of mortality [4, 5]. There is increasing evidence that suggests people living with obesity and obesity‐related chronic conditions are more likely to report loneliness and social isolation than those who are not [6, 7]. This is particularly evident in those with severe and complicated obesity, where its chronic, systemic nature constitutes a greater psychosocial load [8, 9, 10]. Additionally, people living with obesity who are isolated or lonely are at an increased risk of more disease‐specific complications, presence of comorbidities, and mortality, when compared to those not experiencing SIL [6, 7, 11]. Recent research also suggests loneliness can negatively impact weight maintenance following weight loss surgery [12].

Obesity, and particularly severe and complicated obesity, is a risk factor for multiple chronic conditions, including type 2 diabetes mellitus (T2DM), osteoarthritis (OA), nonalcoholic fatty liver disease (NAFLD), metabolic syndrome, and chronic pain, as well as cardiovascular disease (CVD), chronic kidney disease (CKD), and serious mental illness (SMI). Each of these outcomes is more likely with increasing degrees of obesity and collectively can have a significant impact on health and well‐being [13, 14]. However, for these “obesity‐related complications,” the direction of causality in relation to obesity can be unclear. For example, for mental health conditions, the likelihood of being diagnosed increases with increasing body mass index (BMI), whereas the mental health conditions themselves, and the medications used to treat them, may also precipitate weight gain [15, 16].

As well as an increasing degree of obesity, SIL is likely to impact the presence and severity of obesity‐related conditions. Conversely, obesity‐related complications are likely to exacerbate SIL in obesity. For example, social isolation has been significantly associated with rapid decline in kidney function and CKD onset [17]. Loneliness has also been associated with the development of metabolic syndrome, OA, and chronic pain. Conversely, these conditions can lead to SIL [18, 19, 20]. Additionally, SIL can impact the development, progression, and disease outcomes of CVD, mental health conditions, and T2DM [21, 22, 23, 24, 25].

Given the clear links between obesity, obesity‐related complications, and SIL, specific interventions for SIL may have use in the management of obesity and obesity‐related complications to achieve better outcomes. However, there is only limited, heterogeneous literature on the types of interventions and their effects on SIL and/or subsequent obesity/obesity‐related outcomes. A synthesis of available interventions is necessary in informing future interventions in this area, particularly for those with severe and complicated obesity in a practice setting. The treatment of more severe obesity involves clinics with multidisciplinary teams [26, 27], which often function at full capacity, requiring patients to be on long waiting lists prior to entering the program [28]. Long waiting periods may lead to declines in health, motivation, and dropouts, leaving patients without the treatments they require [29, 30]. As previous research has shown these patients to be more socially isolated and lonely, and practical experience has demonstrated that more socially connected patients have better treatment outcomes, addressing SIL in this setting may be of great benefit. Hence, it is important to know the effects of previous interventions to design interventions that may improve outcomes among people with obesity and particularly severe and complicated obesity.

A comprehensive systematic review of SIL interventions for people living with obesity and/or obesity‐related complications was therefore undertaken to synthesize the existing evidence. This review aimed to provide a clearer understanding of the characteristics of existing interventions while assessing their potential impact on SIL among people with obesity and obesity‐related conditions to inform future interventions that may address these interconnected issues among those with severe and complicated obesity.

## 2 Materials and Methods

This systematic review was registered with PROSPERO (CRD42023439710) and follows PRISMA reporting guidelines (Appendix S1) [31, 32].

### 2.1 Search Strategy and Study Selection

An initial scoping search for interventions addressing SIL in people with obesity only resulted in limited findings. Therefore, the search was expanded to include obesity‐related complications, given their relatedness and the impact of SIL on all conditions. A comprehensive search strategy was subsequently developed (Appendix S2) and the databases SCOPUS, PsycINFO, and EMBASE searched for eligible articles for inclusion (May 2023, final search undertaken in December 2024). Search terms included key words from the eligibility criteria including obesity, SIL, and SMI. In addition, reference lists were searched for relevant articles that could also be included.

**FIGURE 1:**
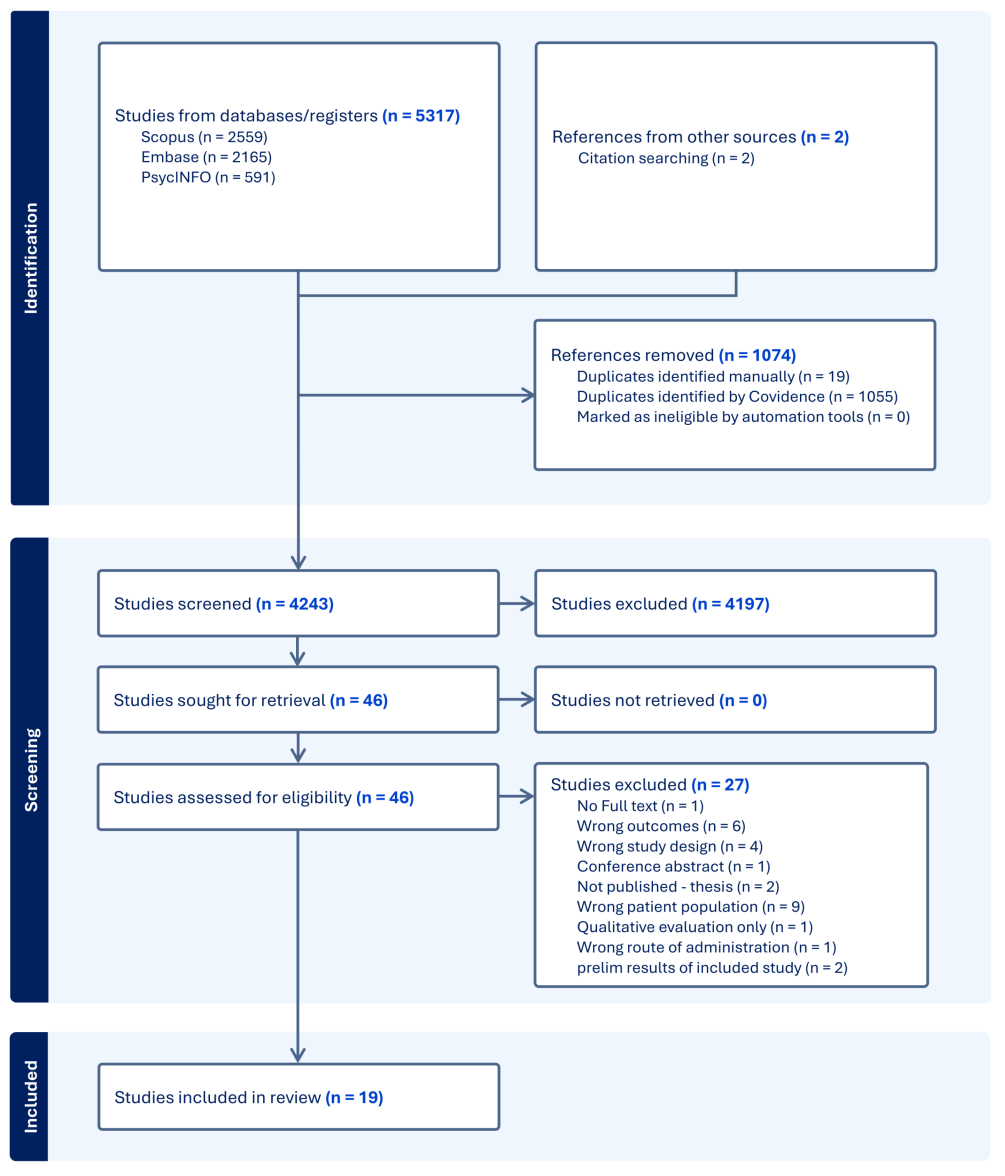
Prisma flow chart somewhere here.

Studies located via the search were uploaded into Covidence, a systematic review management software that was utilized for title and abstract screening, full‐text screening, data extraction, and quality assessment [33]. An initial title and abstract screen were independently undertaken by GA and KM, with disagreements resolved by a third reviewer (MP or KW) (Figure 1). This was followed by a full text screening of potentially eligible studies, which was independently undertaken by GA and KM. Any discrepancies were initially discussed by the two reviewers then with a third review author (KW or MP) when consensus was not reached. Inclusion and exclusion criteria guided study selection with reasons for exclusion noted during full text screening.

### 2.2 Eligibility Criteria

Studies included adult (≥ 18 years) participants with obesity (BMI ≥ 30 kg/m [2]) and/or obesity‐related complications. Based on the literature and the input of authors who are experts in the field (KW and MP), the following obesity‐related complications were deemed to be eligible for inclusion: T2DM, dyslipidaemia, obstructive sleep apnoea (OSA), SMI, OA, CVD (hypertension, coronary heart disease, and heart failure), CKD, chronic pain, and NAFLD [13].

Only intervention studies that included a component to address and evaluate SIL were eligible, including randomized controlled trials (RCTs) and nonrandomized studies (nonrandomized controlled trials and single‐arm pre–post studies). Articles in any language were eligible if an English abstract was available. Dates of studies eligible for inclusion were unlimited.

### 2.3 Data Extraction and Synthesis

Data on study characteristics, SIL and disease‐related outcomes, and intervention components were extracted from included articles. This included study design, study duration, date, setting, delivery method, participant characteristics, details of the intervention and comparison groups, measures used, outcomes and results of the intervention, and intervention design and detail on intervention components. Data extraction was undertaken by GA using Covidence.

Risk‐of‐bias assessments were conducted based on the type of intervention (RCT, nonrandomized trial, or pre–post study). The quality assessment tools utilized were the revised JBI critical appraisal tool for the assessment of risk of bias for randomized controlled trials [34] and the NHLBI before–after (pre–post) studies with no control group assessment [35]. Two reviewers (GA and KM) independently rated the quality of all included studies. Any discrepancies were initially discussed by these two reviewers and then by a third review author (KW or MP) when consensus was not reached.

Data synthesis was performed through a narrative analysis of results. High levels of heterogeneity in intervention types and study outcomes made a meta‐analysis unfeasible.

## 3 Results

### 3.1 Description of Included Studies

Of 3521 studies screened, 19 [36, 37, 38, 39, 40, 41, 42, 43, 44, 45, 46, 47, 48, 49, 50, 51, 52, 53, 54] studies met the inclusion criteria (Figure 1). Studies included 8 RCTs (*n* = 8) [37, 38, 45, 46, 47, 51, 52, 53] and 11 nonrandomized (pre–post) (*n* = 11) [36, 39, 40, 41, 42, 43, 44, 48, 49, 50, 54]. Interventions were primarily conducted in people with SMI (*n* = 9) [36, 37, 40, 42, 44, 45, 47, 49, 52], with the remaining studies focused on CVD (*n* = 3) [38, 50, 54], chronic pain (*n* = 3) [43, 51, 53], T2DM (*n* = 2) [41, 46], obesity (*n* = 1) [39], or both chronic pain and T2DM (*n* = 1) [48]. A table of all data extracted is included in Appendix S3. Of the 19 studies included in this review, six studies reported SIL as a primary outcome [36, 44, 47, 48, 49, 50], whereas the remainder reported SIL as a secondary outcome. Eight used the UCLA loneliness scale [36, 39, 40, 42, 49, 50, 51, 54] to evaluate outcomes, though different versions of the scale were used. The remainder of the studies used a variety of different scales to measure loneliness, whereas six studies evaluated social isolation, with or without loneliness [38, 41, 43, 44, 47, 49] (Table 1).

**TABLE 1:** A table of summary characteristics of included studies.

Study	Study design	Aim of study	Condition/s studied	Scale	SIL result	Other outcomes	Limitations	Effective?
Adery 2022	Pre post test	Primary—loneliness; secondary—symptoms	SMI—schizophrenia‐spectrum conditions (SCZ)	UCLA loneliness scale	Significantly reduced loneliness (*t* [16] = 2.09, *p* = 0.021) with a mean difference score of 6.29 (pre‐M = 30.2, post‐M = 23.8); number of sessions a participant attended was correlated with reduced loneliness (*r* = 4.15, *p* = 0.013).	Significant improvement at postintervention in the reduction of overall psychiatric symptoms BPRS (*t* [16] = 1.89, *p* = 0.032, md = 2.17), depression (BDI) scores (*t* [16] = 2.02, *p* = 0.026, md = 2.05). Nonsignificant changes in SAPS or SANS, PSS, and in CogState	Small sample size, lack of specificity of what is effective due to the number of variables within the intervention itself,	Yes
Ali 2021	Randomized controlled trial	Primary—Depression; secondary—Loneliness	SMI—Depression	The Modified Worker Loneliness Questionnaire (MWLQ)	No statistically significant changes	No statistically significant changes	Issues with recruitment, could not achieve 12 month follow up, self‐reported measures	No
Beauchamp 2024	Pre post test	Primary—feasibility, perceived social support; secondary—loneliness	CVD—Stroke	20 item UCLA loneliness scale	Nonsignificant changes	Nonsignificant changes in social support, depression and anxiety, and self‐efficacy	Results are limited by a small feasibility sample and lack of a control group. Selection bias may have occurred from use of convenience sampling	No
Berkman 2003	Randomized controlled trial	Primary—Mortality, recurrent infarction; secondary—Social Isolation	CVD—Coronary Heart Disease	ENRICHD Social Support Instrument (ESSI)	ESSI score from baseline were significantly higher in the intervention than the usual care group (24.4 vs. 22.6 and 27% vs. 18%, respectively).	No significant difference between treatments in recurrence of MI or death (log‐rank *p* = 0.94).	6‐month time frame may be insufficient to address social support	Yes
Forbes 2020	Pre post test	Primary—weight stigma; secondary—Loneliness	Overweight and obesity	UCLA loneliness scale	Significant improvement in loneliness from preintervention to postintervention.	Significant improvement in self‐compassion and internalized weight stigma with a large effect size. Significant improvements in psychological distress, life satisfaction, body dissatisfaction, body shame, and eating self‐efficacy. Nonsignificant trend of mean group weight loss.	Preliminary results, small sample of Caucasian female participants who were self‐selected, which limits generalizability.	Yes
Fortuna 2022	Pre post test	Primary—feasibility, self‐management; secondary—Loneliness	SMI	20‐item UCLA Loneliness Scale	No statistically significant improvement in loneliness.	Significant increase in self‐efficacy to manage the chronic disease and Empowerment. No statistically significant changes in psychiatric self‐management, medical self‐management.	Pilot, so small sample size so not powered to detect statistically significant pre/post differences, no control group	No
Ghahari 2015	Pre post test	Primary—diabetes self‐management; secondary—SIL	Diabetes	Scale for social isolation Duke Loneliness Scale	No statistically significant changes in loneliness and social isolation between groups or at different time points.	Significantly more people in the Chronic Disease program made clinically important improvements in their self‐efficacy than did those in the Diabetes program	Self‐reported, no control group	No
Hoy‐Gerlach 2022	Pre post test	Primary—mental health recovery; secondary—loneliness	SMI	UCLA	Statistically significant decreases in UCLA Loneliness Scale scores from Time 1 (M = 59.20, SD = 9.47) to Time 2 [M = 49.90, SD = 13.66, *t*(10) = 3.80, *p* = 0.004]. The eta‐squared statistic (0.62) indicated a large effect size.	Statistically significant decrease in Becks Depression Inventory (BDI) total scores from Time 1 (M = 21.09, SD = 8.43) to Time 2 [M = 14.64, SD = 7.03, *t*(10) = 2.48, *p* = 0.03], and statistically significant decrease in Becks Anxiety Inventory (BAI) scores from Time 1 (M = 23.55, SD = 9.81) to Time 2 [M = 17.73, SD = 11.79, *t*(10) = 2.24, *p* = 0.049].	Findings are not generalizable; not a randomly selected or assigned sample, no control or comparison group	Yes
Koebner 2019	Pre post test	Primary—feasibility, chronic pain; secondary—Social Isolation	Chronic pain	12‐item social disconnection scale	Participants had higher preintervention social disconnection scores (M = 26.00, SD = 9.86) than postintervention (M = 22.35, SD = 9.86). This difference represented a moderate effect size (d = 0.37).	On average, participants had higher pre intervention pain unpleasantness scores (M = 4.02, SD = 2.42) than postintervention (M = 3.53, SD = 2.61). This difference represented a small effect size (*d* = 0.20). Changes in pain intensity pretour to 3‐week follow‐up (pre = 4.14, SD = 2.24; 3‐week M = 3.51, SD = 2.48; difference = 0.63, BCa 95% CI = 0.07–1.25, *p* = 0.034, *d* = 0.28)	Small sample size, lack of comparison group and randomization, self‐reported, selection bias.	Yes
Loi 2016	Pre post test	Primary—social isolation. Self esteem	SMI	Hawthorne Friendship scale	No statistical differences	No statistical differences in self‐esteem, friendship, or in the internet questionnaires.	Very small sample size, no control	No
Muralidharan 2020	Randomized controlled trial	Primary—health related quality of life; secondary—loneliness	SMI	Three‐Item Loneliness Scale	In‐person MOVE was associated with a greater decrease in the Three Item Loneliness Scale total score at 6 months (*t* = 2.76, *p* = 0.006).	There were significant increases in both active interventions in VR‐12 MCS compared to usual care: for WebMOVE, at three months (*t* = 2.17, *p* = 0.031) and 6 months (*t* = 2.38, *p* = 0.018), and for in‐person MOVE at 6 months (*t* = 1.99, *p* = 0.048). No significant group differences on the BASIS scales, General Life Satisfaction, IWQOL‐PF, or VR‐12 PCS.	Participants were Veterans and mostly males; thus, findings may not generalize to other populations.	Yes
Saghaee 2020	Randomized controlled trial	Primary—self efficacy; secondary—loneliness	Diabetes	Adult Social Emotional Loneliness Scale‐Short form (SELSA‐S)	No significant change in the control and intervention groups in terms of loneliness after controlling for baseline data (*p* = 0.75).	Significant improvement in the medical self‐efficacy domain, for the intervention group as compared to the control group (*p* = 0.02). No significant differences between the control and intervention groups after the four‐week follow‐up for PHQ9 (*p* = 0.38)	Small sample size, differences at baseline	No
Shih 2023	Randomized controlled trial	Primary—social isolation, quality of life	SMI‐ schizophrenia	Mental health‐social functioning scale (MHSFS)	No statistically significant differences between groups	No statistically significant differences between groups in WHOQOL scores.	Small sample size	No
Smith 2022	Pre post test	Primary—loneliness	Chronic disease ‐ sub analysis for diabetes and chronic pain	The Campaign to End Loneliness Measurement Tool (CEL)	Significant differences were found loneliness scores improved from baseline to 6 weeks (3.16 ± 3.16 vs. 2.08 ± 2.37, respectively) in the unadjusted model (*p* < 0.001) and the model adjusting for participant and workshop characteristics (*p* < 0.001).	NA	Self‐reported, no comparison group, short time frame (6 weeks)	Yes
Steinman 2021	Pre post test	Primary—social connectedness (including SIL)	SMI‐ Depression	Duke Social Support Index (DSSI‐10) Patient‐Reported Outcomes Measurement Information System Social Isolation (PROMIS‐SI) 3 item UCLA‐Loneliness	All three scales showing statistically significant (*p* < 0.001) changes at follow‐up using paired t‐tests (DSSI‐10: *t* = 5.2, df = 312, *p* < 0.001; PROMIS: *t* = 6.3, df = 310, p < 0.001; UCLA: *t* = 3.7, df = 301, *p* < 0.002).	Reduced depression, the mean (SD) change in depression (PHQ‐ 9) was 5.4 (5.3) pre–post PEARLS	No comparison group or randomization,	Yes
Theeke 2021	Pre post test	Primary—feasibility, loneliness	CVD ‐ Stroke	Loneliness (Revised UCLA Loneliness Scale)	Mean loneliness changed significantly (*t* = 2.744, *p* = 0.04) from1 week post last LISTEN session (mean 57.17, SD 6.24) to12 weeks post last LISTEN session (mean = 51.83, SD 3.43) indicating participants continued to become less lonely	No significant changes in mean depression scores, Neuro QoL. Mean PHQ‐9 scores diminished significantly from 6–12 weeks post LISTEN (*t* = 2.52, *p* = 0.053). Mean systolic blood pressure decreased from an average of 149 (SD 30.62) to a mean of 135 (SD 19.86) which is clinically relevant in stroke survivors	Small sample size	Yes
Tse 2014	Randomized controlled trial	Primary—pain management; secondary—loneliness	Chronic pain	Chinese version of the UCLA Loneliness Scale	Loneliness levels dropped significantly in both groups (from 44.5 + _ 8.7 to 34.3 + _ 8.3) (*p* < 0.001) and from 42.8 6 10.5 to 38.9 + _ 9.8 (*p* = 0.031). No significant differences between the two groups at both baseline and at week 12 (*p* > 0.05)	Nonsignificant pain reduction between groups	Self‐reported data, small sample size	No
Tse 2023	Randomized controlled trial	Primary—pain intensity; secondary—loneliness	Chronic pain	Chinese version of the 6‐item De Jong Gierveld Loneliness Scale (DJGLS)	No significant changes when compared to control	Significant reduction in pain intensity when compared to control, but not pain self‐efficacy or depression	Limited sample size	No
van Gestel‐Timmermans 2012	Randomized controlled trial	Primary—recovery; secondary—loneliness	Serious mental illness	11 item de jong Loneliness Scale	No effect was found on loneliness	The effect of the intervention (Cohen's *d*) was small to moderate on empowerment and hope and small on self‐efficacy beliefs, quality of life.	Unclear precisely which ingredients contributed to the effect of the peer‐run course and how often participants should attend the course to benefit from it	No

Interventions were either group based (*n* = 10) [36, 39, 41, 43, 46, 47, 48, 50, 51, 52, 53, 54] or focused on sessions or activities for the individual (*n* = 5) [37, 40, 42, 44, 49]. Two interventions were a mixture of group‐based and individual activities [38, 45]. Studies were further stratified as being conducted in person (*n* = 15) [36, 37, 38, 39, 41, 42, 43, 46, 47, 48, 49, 50, 51, 52, 53], virtually (*n* = 3) [40, 44, 54], or both (*n* = 1) [45].

Studies were also categorized into four groups based upon the predominant intervention type of the study. This comprised of therapeutic, such as therapy, education, or training (*n* = 12) [38, 39, 40, 41, 44, 46, 48, 49, 50, 51, 52, 54]; companionship, such as befriending or animal companionship (*n* = 3) [37, 42, 47]; physical type, such as exercise or singing (*n* = 3) [36, 45, 53]; and social activity, such as a guided group tour (*n* = 1) [43] based interventions. The components of these interventions were often to provide social support, promote social interaction, provide social cognition training and/or social skills training. Studies included had at least one, or a combination, of these components to address loneliness and/or social isolation (Table 1).

### 3.2 Effect of Interventions on SIL

Most studies showing efficacy in reducing SIL were nonrandomized pre–post studies, with 7/11 (64%) reporting significant reductions in SIL from baseline to postintervention [36, 39, 42, 43, 48, 49, 50]. Among the RCTs, only two of eight studies (25%) found significant reductions in SIL compared to the controls [38, 45].

Interventions that incorporated group activities and opportunities for shared experiences mostly reported significant reductions in loneliness and social isolation, whereas individual and technology‐based interventions were more likely to report nonsignificant results in addressing SIL. Most studies were therapeutic in nature, though it is unclear how this impacted on SIL outcomes.

#### 3.2.1 Interventions for SIL Among People With Obesity

Only one pre–post study was found to address loneliness specifically in people with obesity [39]. This study included 15 women (mean age 43.6 [12.38]) with either overweight or obesity (mean BMI 31.23 [5.8]) and utilized compassion focused therapy (CFT) to address weight stigma, with a secondary aim of increasing self‐compassion, reducing loneliness, and improving other psychosocial factors in participants. The primary component of this intervention, delivered via an intensive 2‐week program (five sessions per week), was social cognitive training and promotion of social interaction via a group. The authors hypothesized that social cognitive training through CFT reduced internalized barriers, whereas the group‐based nature of the intervention fostered a sense of shared experience and camaraderie among participants. The intervention showed significant reductions in loneliness on the eight‐item UCLA loneliness scale from preintervention to postintervention (13.47 [4.73] to 10.2 [5.75], *p* < 0.001), but this was not maintained at 3 months follow‐up. However, improvements in self‐compassion and weight stigma were maintained at follow up [39].

#### 3.2.2 Interventions for SIL and Obesity‐Related Mental Health Conditions

SIL interventions for individuals with a SMI, including schizophrenia, bipolar disorder, and major depressive disorder, showed varying degrees of success [36, 37, 40, 42, 44, 45, 47, 49, 52]. Of the nine studies included that focussed on individuals with SMI, four reported significant reductions in SIL [36, 42, 45, 49], of which one was an RCT [45].

Of these four interventions, two were physical‐type interventions (*singing and exercise*) [36, 45]. One of these was an RCT comparing an in‐person and web‐based exercise program with usual care (standard services and weight management information) on SMI participants with weight issues (mean age 81 [9.4], BMI 34.5 [5.3]). Only the in‐person group showed significant reductions in loneliness on the three‐item UCLA loneliness scale at 6 months (*t* = −2.76, *p* = 0.006) [45]. The other physical type intervention was a group based, in‐person choir study, with pre–post design [36]. The remaining two pre–post studies were both in‐person studies for individuals, of which one was a companion‐based intervention [42, 49].

For studies that did not show any efficacy in reducing SIL, two were individual, technology‐based therapeutic interventions [40, 44]. Additionally, two companion‐based interventions, despite being in person, were reported to be ineffective [37, 47], of which one was an RCT [37]. Finally, an RCT group‐based, in‐person, therapeutic intervention utilizing peer instructors reported no observed effect on loneliness [52].

#### 3.2.3 Interventions for SIL and Obesity‐Related Physical Health Conditions

Studies focusing on people with CVD included participants postmyocardial infarction and stroke. Two studies, including one RCT [38], both employing in‐person, group therapeutic interventions (activities, education, and therapy), reported efficacy in reducing loneliness and social isolation [38, 50], whereas a virtual environment‐based pilot study did not [54].

Two interventions for participants experiencing chronic disease with chronic pain demonstrated positive outcomes in reducing social isolation and loneliness [43, 48]. Of these, one intervention was a pilot social activity intervention, whereas the other was a therapeutic intervention, which employed a chronic disease self‐management program (CDSMP). This study reported reduction in loneliness for both chronic pain and T2DM [48]. However, a therapeutic peer‐led pain management group program and a physical type group intervention, one in nursing home residents and one in community dwelling older adults, both of which were RCTs, reported that both intervention and control groups experienced significant reductions in loneliness post intervention, with no significant difference between intervention and control groups [51, 53]. The control groups received either 1‐h pain management from the research team for the duration of the study [51] or usual care in addition to a pain management pamphlet [53].

Interventions for participants with T2DM showed mixed results in addressing loneliness, despite all studies utilizing some form of CDSMP [41, 46, 48]. The intervention that reported effectiveness in loneliness reduction (in both T2DM and chronic pain) was a pre–post study with loneliness as the primary outcome of the intervention [48]. Of the two studies that showed no significant changes, one was an RCT [46] and the other a pre–post study [41], with both studies investigating SIL as a secondary outcome.

#### 3.2.4 Effects of Interventions on Other Physical and Psychosocial Outcomes

Some studies also reported improvement in disease‐related outcomes, including psychiatric symptoms [36, 42, 49], mean systolic blood pressure [50], and pain [43, 51, 53]. All except for one [51] of these studies also reported significant reductions in SIL. Other studies reported no improvement in disease‐related outcomes such as weight loss [39], depressive symptoms [37], and CVD‐related outcomes [38], despite two reporting improvements in SIL [38, 39].

Studies also reported improvement in other psychosocial outcomes including self compassion [39], weight stigma [39], quality of life [45, 46, 47, 52], self‐efficacy [40, 41, 46], empowerment [52], and self‐care [46], whereas others reported no significant improvement in self‐esteem [44] and other psychosocial factors [53, 54].

### 3.3 Quality Assessment and Risk of Bias

None of the included studies, RCT or otherwise, fulfilled the criteria required to be a high‐quality study. For the nine RCTs, based on the utilization of the JBI assessment tool, five studies had a low risk of bias [37, 46, 47, 51, 53], whereas the remainder of the studies were of moderate risk (Appendix S4) [38, 45, 52]. Issues included a lack of blinding, as well as unclear reliability around outcomes measured.

For the 10 nonrandomized pre–post studies, based on the utilization of the NHLBI assessment tool, all studies were rated as fair, indicating a moderate risk of bias (Appendix S5) [36, 39, 40, 41, 42, 43, 44, 49, 50, 54]. Issues included a lack of reporting on sufficiency of sample size, blinding, and frequency of measures. However, we note that all nonrandomized studies are more likely to be biased compared to RCTs, with pre–post studies inherently biased due to the lack of a control group.

## 4 Discussion

This is the first study to systematically review interventions aimed at reducing SIL in individuals with obesity and/or obesity‐related complications. Despite a robust search, we were only able to identify a limited number of studies (*n* = 19, 8 RCTs, 11 pre–post) addressing SIL in obesity and/or obesity‐related complications, with only one intervention specifically targeting individuals with obesity, and no studies for several other obesity‐related complications (dyslipidaemia, OSA, OA, CKD, and NAFLD). Most of the included studies (*n* = 9) focused on SMI; an expected finding due to greater awareness of psychosocial variables in mental health conditions [23, 24]. This concentration on SMI is particularly valuable for obesity research given the bidirectional relationship between SMI and obesity, as evidenced by one SMI intervention which also incorporated a weight management component [45]. Despite this, the strength of evidence for findings is not strong, as most studies included are pre–post studies of moderate quality. This is particularly relevant in relation to studies reported as effective, as only 25% of RCTs were effective in reducing SIL (vs. 64% of pre–post studies). Although the limited research aligns with the emerging nature of this field of inquiry, it emphasizes the need for more research, especially in developing targeted and robust SIL interventions for individuals with obesity and obesity‐related complications.

In addition to some reductions being seen in SIL, there were some reported improvements in a range of other psychosocial and physiological outcomes, with most studies demonstrating improvement in disease‐specific outcomes also reporting improvements in SIL. These studies tended to be more intensive and of longer duration, consistent with current literature in addressing disease‐related factors [55]. This may be due to the cyclical relationship between physiological and psychosocial health, as physical limitations and suboptimal self‐care can lead to stigma and fewer social networks, and vice versa [56]. However, this relationship is complex and can be influenced by other factors, with two studies showing an improvement in SIL without corresponding improvements in disease‐related factors. This suggests that addressing SIL does not necessarily lead to improvements in disease‐specific outcomes. This complex relationship warrants further investigation, and future studies should aim to elucidate the underlying mechanisms between SIL interventions and health outcomes in obesity and obesity‐related complications with robust obesity‐focused trials. Such research could potentially inform more targeted and effective intervention strategies, addressing both SIL and disease‐specific factors in this population.

The design and delivery of interventions targeting SIL in obesity and obesity‐related complications varied considerably across the reviewed studies. Most interventions were therapeutic in nature, aiming to address both disease‐specific outcomes and SIL. This approach, although logical, may also benefit from incorporating more sustained social components in future designs as previously suggested [57]. In person, group‐based approaches appeared to have the most efficacy overall, though some individual in‐person interventions also reported positive outcomes. However, this trend was not universal; in two animal companionship studies, one individual‐based intervention [42] proved effective, whereas the group‐based [47] one did not, suggesting that some intervention types may be more appropriate in an individual setting. Consistent with existing literature, this finding suggests that although group‐based interventions may be more useful for SIL, the reality is more nuanced and, for some, an individual approach may be preferred, with the sustainability of the intervention more likely to determine effectiveness [58]. This is particularly relevant for individuals with severe and complicated obesity who are likely to experience societal stigma and lower self‐esteem, which can lead to a reluctance to participate in group‐based activities, at least initially [8, 59]. Consideration must be given to these potential challenges when designing interventions for individuals with obesity and obesity‐related conditions, by incorporating components that address potential barriers, and opting for a stepped approach for those who are extremely isolated [9]. From a clinical perspective, management of severe and complicated obesity has benefited from supportive components, whether in‐person individual or supervised group‐based activities, and has been shown to complement diet, lifestyle, and therapeutic interventions by promoting physical and psychosocial wellbeing, and contributing to weight loss and maintenance, particularly postbariatric surgery [60, 61, 62].

Remote, technology‐based interventions did not demonstrate efficacy in reducing SIL [40, 44, 54]. This may, in part, be due to the challenge of maintaining sustained engagement necessary for ongoing reductions in SIL. Human connection can also facilitate communication, with technological interventions unlikely able to provide the depth of support needed to reduce SIL [63]. Lack of personalization is therefore likely to reduce effectiveness for individuals with more complex needs, such as people with severe and complicated obesity, with technological interventions combined with some human interaction most likely to be effective to address both weight loss and SIL [64]. Further, there was significant heterogeneity among technology‐based SIL interventions, with wide variation in the design, delivery, and content. It is therefore challenging to identify which components could be the most beneficial for addressing SIL with further research in this area warranted, especially for physically isolated individuals, such as those with severe and complicated obesity [65].

This review found several methodological challenges and areas for improvement in SIL intervention research for obesity and related conditions, with the evidence being low‐moderate in quality overall. These include the difficulty in assessing chronic loneliness, as loneliness can be transient in nature, especially in short‐duration studies; inconsistent intervention efficacy compared to control conditions [51, 53], suggesting either varying effectiveness or beneficial factors in both intervention and control groups; difficulty comparing study designs with SIL as a primary outcome [48], to those where it is a secondary outcome [41, 46], due to potentially confounding factors; and a predominance of pre–post study designs with significant heterogeneity in intervention characteristics and SIL measurement methods, indicating a lack of consensus on effective approaches. These findings highlight the need for more rigorous and standardized research approaches to intervening to improve SIL, including careful selection of control conditions and consideration of longer term outcomes. Although existing frameworks can be utilized [57], there is room for innovation in study design, such as incorporating community development approaches involving service users in intervention design and implementation [66]. Future research addressing these limitations could significantly enhance our understanding of effective SIL interventions in obesity and related conditions.

## 5 Conclusion

This systematic review highlights that despite some promising evidence, there is a critical need for more targeted research on social isolation and loneliness interventions for individuals with obesity, particularly those with severe and complicated obesity as well as obesity‐related diseases. Future studies should focus on longer duration interventions, standardized measurement techniques, and innovative designs that consider both physical and psychological aspects of obesity and SIL.

## Conflicts of Interest

The authors declare no conflicts of interest.

## Supporting information


**Data S1:** Supplementary Information.

## Data Availability

The data that support the findings of this study are available in the Supporting Information.

## References

[1] D. Ding , R. Eres , and D. L. Surkalim , “A Lonely Planet: Time to Tackle Loneliness as a Public Health Issue,” BMJ: British Medical Journal (Online) 377 (2022): o1464. 10.1136/bmj.o1464 35700988

[2] M.‐H. Hall , Y. Xiao , D. Öngür , J. Torous , and D. V. Jeste , “Social Isolation and Loneliness: Modern Pandemic of a Psychosocial Determinant of Health,” Psychiatric Annals 54, no. 7 (2024): e196–e201, 10.3928/00485713-20240618-01.

[3] C. M. Jarach , M. Tettamanti , A. Nobili , and B. D'Avanzo , “Social Isolation and Loneliness as Related to Progression and Reversion of Frailty in the Survey of Health Aging Retirement in Europe (SHARE),” Age and Ageing 50, no. 1 (2021): 258–262, 10.1093/ageing/afaa168. 32915990 PMC7793602

[4] S. Shiovitz‐Ezra and L. Ayalon , “Situational Versus Chronic Loneliness as Risk Factors for All‐Cause Mortality,” International Psychogeriatrics 22, no. 3 (2010): 455–462, 10.1017/S1041610209991426. 20003631

[5] X. Wang , H. Ma , X. Li , Y. Heianza , V. Fonseca , and L. Qi , “Joint Association of Loneliness and Traditional Risk Factor Control and Incident Cardiovascular Disease in Diabetes Patients,” European Heart Journal 44 (2023): ehad306, 10.1093/eurheartj/ehad306. PMC10361009 37385629

[6] T. Petitte , J. Mallow , E. Barnes , A. Petrone , T. Barr , and L. Theeke , “A Systematic Review of Loneliness and Common Chronic Physical Conditions in Adults,” Open Psychology Journal 8, no. 1 (2015): 113–132, 10.2174/1874350101508010113. 26550060 PMC4636039

[7] J. Zhou , R. Tang , X. Wang , X. Li , Y. Heianza , and L. Qi , “Improvement of Social Isolation and Loneliness and Excess Mortality Risk in People With Obesity,” JAMA Network Open 7, no. 1 (2024): e2352824, 10.1001/jamanetworkopen.2023.52824. 38252435 PMC10804268

[8] J. Termaat , M. K. Piya , and K. A. McBride , “Community‐Based Care Needs for Adults With Class III Obesity Before and After Tertiary Weight Management: An Exploratory Study,” Obesity Science and Practice 10, no. 1 (2024): e732, 10.1002/osp4.732. 38213316 PMC10782639

[9] G. Alsultany , A. el Masri , F. MacMillan , K. Williams , and K. McBride , “Support Needs of People Living With Obesity During Transition From Tertiary Obesity Treatment to Community Care,” Obesity Research & Clinical Practice 16, no. 6 (2022): 514–523, 10.1016/j.orcp.2022.09.005. 36207249

[10] F. Rubino , D. E. Cummings , R. H. Eckel , et al., “Definition and Diagnostic Criteria of Clinical Obesity,” Lancet Diabetes & Endocrinology 13, no. 3 (2025): 221–262, 10.1016/S2213-8587(24)00316-4. 39824205 PMC11870235

[11] S. Atasoy , K. H. Ladwig , J. Kruse , K. Lukaschek , and A. Peters , “Inverse Relationship Between Social Isolation and Type 2 Diabetes Incidence in People With Obesity: Findings From the MONICA/KORA Prospective Cohort,” Journal of Psychosomatic Research 121 (2019): 124. Seventh Annual Scientific Conference of the European Association of Psychosomatic Medicine (EAPM). Rotterdam Netherlands, 10.1016/j.jpsychores.2019.03.076.

[12] M. Bezuidenhout , “An Exploration of Psychological Factors That Contribute to Weight Maintenance After Weight Loss Surgery,” Dissertation Abstracts International 85, no. B (2024): 6‐B.

[13] P. E. O'Brien and J. B. Dixon , “The Extent of the Problem of Obesity,” American Journal of Surgery 184, no. 6 (2002): S4–S8. 10.1016/s0002-9610(02)01172-8 12527343

[14] J. Moini , R. Ahangari , C. Miller , and M. Samsam , “Chapter 18—Perspective on Economics and Obesity,” in Global Health Complications of Obesity, eds. J. Moini , R. Ahangari , C. Miller , and M. Samsam (Elsevier, 2020), 411–423.

[15] L. J. Ells , R. Lang , J. P. Shield , et al., “Obesity and Disability—A Short Review,” Obesity Reviews 7, no. 4 (2006): 341–345. 17038128 10.1111/j.1467-789X.2006.00233.x

[16] V. H. Taylor , M. Forhan , S. N. Vigod , R. S. McIntyre , and K. M. Morrison , “The Impact of Obesity on Quality of Life,” Best Practice & Research. Clinical Endocrinology & Metabolism 27, no. 2 (2013): 139–146. 23731876 10.1016/j.beem.2013.04.004

[17] R. N. Moorthi and K. Latham‐Mintus , “Social Isolation in Chronic Kidney Disease and the Role of Mobility Limitation,” Clinical Kidney Journal 12, no. 4 (2019): 602–610, 10.1093/ckj/sfy134. 31384455 PMC6671555

[18] J. M. Wilson , C. A. Colebaugh , S. M. Meints , K. Mikayla Flowers , R. R. Edwards , and K. L. Schreiber , “Loneliness and Pain Catastrophizing Among Individuals With Chronic Pain: The Mediating Role of Depression,” Journal of Pain Research 15 (2022): 2939–2948, 10.2147/JPR.S377789. 36147455 PMC9488611

[19] C. Zheng , M. H. He , J. R. Huang , and Y. He , “Causal Relationships Between Social Isolation and Osteoarthritis: A Mendelian Randomization Study in European Population,” International Journal of General Medicine 14 (2021): 6777–6786, 10.2147/IJGM.S331864. 34703283 PMC8523904

[20] A. de la Torre‐Luque , E. Lara , J. de la Fuente , et al., “Metabolic Dysregulation in Older Adults With Depression and Loneliness: The ATHLOS Study,” Psychoneuroendocrinology 123 (2021): 123104918, 10.1016/j.psyneuen.2020.104918. 33113390

[21] N. K. Valtorta , M. Kanaan , S. Gilbody , S. Ronzi , and B. Hanratty , “Loneliness and Social Isolation as Risk Factors for Coronary Heart Disease and Stroke: Systematic Review and Meta‐Analysis of Longitudinal Observational Studies,” Heart (British Cardiac Society). 102, no. 13 (2016): 1009–1016. Original article., 10.1136/heartjnl-2015-308790. 27091846 PMC4941172

[22] R. M. Long , A. Terracciano , A. R. Sutin , et al., “Loneliness, Social Isolation, and Living Alone Associations With Mortality Risk in Individuals Living With Cardiovascular Disease: A Systematic Review, Meta‐Analysis, and Meta‐Regression,” Psychosomatic Medicine 85, no. 1 (2023): 8–17, 10.1097/PSY.0000000000001151. 36441849

[23] E. Erzen and Ö. Çikrikci , “The Effect of Loneliness on Depression: A Meta‐Analysis,” International Journal of Social Psychiatry 64, no. 5 (2018): 427–435, 10.1177/0020764018776349. 29792097

[24] J. Wang , F. Mann , B. Lloyd‐Evans , R. Ma , and S. Johnson , “Associations Between Loneliness and Perceived Social Support and Outcomes of Mental Health Problems: A Systematic Review,” BMC Psychiatry 18 (2018): 156, 10.1186/s12888-018-1736-5. 29843662 PMC5975705

[25] R. E. Henriksen , R. M. Nilsen , and R. B. Strandberg , “Loneliness Increases the Risk of Type 2 Diabetes: A 20 Year Follow‐Up—Results From the HUNT Study,” Diabetologia 66, no. 1 (2023): 82–92, 10.1007/s00125-022-05791-6. 36168066 PMC9729154

[26] T. C. Osmundsen , U. Dahl , and B. Kulseng , “Enhancing Knowledge and Coordination in Obesity Treatment: A Case Study of an Innovative Educational Program,” BMC Health Services Research 19, no. 1 (2019): 278, 10.1186/s12913-019-4119-9. 31046766 PMC6498688

[27] National Association of Clinical Obesity Services , “National Framework for Clinical Obesity Services,” (2020).

[28] E. Atlantis , N. Kormas , K. Samaras , et al., “Clinical Obesity Services in Public Hospitals in Australia: A Position Statement Based on Expert Consensus,” Clinical Obesity 8, no. 3 (2018): 203–210, 10.1111/cob.12249. 29683555

[29] I. E. Jepsen , K. B. Petersen , M. L. M. Wissing , T. V. F. Hviid , N. S. Macklon , and A. L. M. Englund , “Turning Waiting Time Into Treatment Time: Weight Reduction by a Lifestyle Intervention Programme for Patients With Obesity Before Fertility Treatment,” Reproductive, Female and Child Health 2, no. 3 (2023): 133–142, 10.1002/rfc2.46.

[30] P. Hanson , C. Summers , A. Panesar , et al., “Implementation of a Digital Health Tool for Patients Awaiting Input From a Specialist Weight Management Team: Observational Study,” JMIR Human Factors 10 (2023): e41256, 10.2196/41256. 37256653 PMC10267795

[31] M. J. Page , J. E. McKenzie , P. M. Bossuyt , et al., “The PRISMA 2020 Statement: An Updated Guideline for Reporting Systematic Reviews,” BMJ 372 (2021): n71, 10.1136/bmj.n71. 33782057 PMC8005924

[32] PROSPERO , “A Systematic Review of Interventions Addressing Loneliness and Social Isolation in People Living With Obesity and Common Comorbid Conditions,” https://www.crd.york.ac.uk/prospero/display_record.php?ID=CRD42023439710.

[33] Innovation VH , “Covidence Systematic Review Software,” http://www.covidence.org.

[34] T. H. S. J. Barker , K. Sears , M. Klugar , et al., “The Revised JBI Critical Appraisal Tool for the Assessment of Risk of Bias for Randomized Controlled Trials,” JBI Evidence Synthesis 21, no. 3 (2023): 494–506. 36727247 10.11124/JBIES-22-00430

[35] National Heart, Lung, and Blood Institute , “Quality Assessment Tool for Beforeafter (Pre‐Post) Studies With No Control Group,” date accessed 2024, http://www.nhlbi.nih.gov/health‐pro/guidelines/in‐develop/cardiovascularrisk‐reduction/tools/before‐after.

[36] L. H. Adery and S. Park , “A Pilot Choral Intervention in Individuals With Schizophrenia‐Spectrum Conditions; Singing Away Loneliness,” Psychiatry Journal 11, no. 2 (Apr 2022): 227–231, 10.1002/pchj.527. 35196745

[37] A. Ali , E. McKenzie , A. Hassiotis , et al., “A Pilot Randomised Controlled Trial of Befriending by Volunteers in People With Intellectual Disability and Depressive Symptoms,” Journal of Intellectual Disability Research 65, no. 11 (2021): 1010–1019, 10.1111/jir.12886. 34570405 PMC9291894

[38] L. F. Berkman , J. Blumenthal , M. Burg , et al., “Effects of Treating Depression and Low Perceived Social Support on Clinical Events After Myocardial Infarction: The Enhancing Recovery in Coronary Heart Disease Patients (ENRICHD) Randomized Trial,” Journal of the American Medical Association 289, no. 23 (2003): 3106–3116, 10.1001/jama.289.23.3106. 12813116

[39] Y. N. Forbes , R. L. Moffitt , M. van Bokkel , and C. L. Donovan , “Unburdening the Weight of Stigma: Findings From a Compassion‐Focused Group Program for Women With Overweight and Obesity,” Journal of Cognitive Psychotherapy 34, no. 4 (2020): 336–357, 10.1891/JCPSY-D-20-00015. 33372127

[40] K. L. Fortuna , A. L. Myers , J. Ferron , et al., “Assessing a Digital Peer Support Self‐Management Intervention for Adults With Serious Mental Illness: Feasibility, Acceptability, and Preliminary Effectiveness,” Journal of Mental Health 31, no. 6 (2022): 833–841, 10.1080/09638237.2021.2022619. 35088619 PMC9329481

[41] S. Ghahari , T. Packer , D. Boldy , L. Melling , and R. Parsons , “Comparing Effectiveness of Generic and Disease‐Specific Self‐Management Interventions for People With Diabetes in a Practice Context,” Canadian Journal of Diabetes 39, no. 5 (2015): 420–427, 10.1016/j.jcjd.2015.04.007. 26145484

[42] J. Hoy‐Gerlach , A. Vincent , B. Scheuermann , and M. Ojha , “Exploring Benefits of Emotional Support Animals (ESAs): A Longitudinal Pilot Study With Adults With Serious Mental Illness (SMI),” Human‐Animal Interaction Bulletin 10, no. 2 (2022): 1–19, 10.1079/hai.2022.0016.

[43] I. J. Koebner , S. M. Fishman , D. Paterniti , et al., “The Art of Analgesia: A Pilot Study of Art Museum Tours to Decrease Pain and Social Disconnection Among Individuals With Chronic Pain,” Pain Medicine 20, no. 4 (2019): 681–691, 10.1093/pm/pny148. 30053185

[44] S. M. Loi , S. Hodson , D. Huppert , J. Swan , A. Mazur , and N. T. Lautenschlager , “Can a Short Internet Training Program Improve Social Isolation and Self‐Esteem in Older Adults With Psychiatric Conditions?,” International Psychogeriatrics 28, no. 10 (2016): 1737–1740, 10.1017/S1041610216001022. 27373436

[45] A. Muralidharan , C. H. Brown , Y. Zhang , et al., “Quality of Life Outcomes of Web‐Based and In‐Person Weight Management for Adults With Serious Mental Illness,” Journal of Behavioral Medicine 43, no. 5 (2020): 865–872, 10.1007/s10865-019-00117-1. 31741204 PMC7231669

[46] A. Saghaee , S. Ghahari , E. Nasli‐Esfahani , F. Sharifi , M. Alizadeh‐Khoei , and M. Rezaee , “Evaluation of the Effectiveness of Persian Diabetes Self‐Management Education in Older Adults With Type 2 Diabetes at a Diabetes Outpatient Clinic in Tehran: A Pilot Randomized Control Trial,” Journal of Diabetes and Metabolic Disorders 19, no. 2 (2020): 1491–1504, 10.1007/s40200-020-00684-0. 33520849 PMC7843754

[47] C. A. Shih and M. H. Yang , “Effect of Animal‐Assisted Therapy (AAT) on Social Interaction and Quality of Life in Patients With Schizophrenia During the COVID‐19 Pandemic: An Experimental Study,” Asian Nursing Research 17, no. 1 (2023): 37–43, 10.1016/j.anr.2023.01.002. 36646276 PMC9837379

[48] M. L. Smith , E. Chen , C. A. Lau , D. Davis , J. W. Simmons , and A. L. Merianos , “Effectiveness of Chronic Disease Self‐Management Education (CDSME) Programs to Reduce Loneliness,” Chronic Illness 19 (2022): 646–664, 10.1177/17423953221113604. 35957597

[49] L. Steinman , A. Parrish , C. Mayotte , et al., “Increasing Social Connectedness for Underserved Older Adults Living With Depression: A Pre‐Post Evaluation of PEARLS,” American Journal of Geriatric Psychiatry 29, no. 8 (2021): 828–842, 10.1016/j.jagp.2020.10.005. PMC7564120 33187883

[50] L. A. Theeke , J. A. Mallow , and E. Theeke , “A Pilot One Group Feasibility, Acceptability, and Initial Efficacy Trial of LISTEN for Loneliness in Lonely Stroke Survivors,” SAGE Open Nursing 7 (2021): 1–8, 10.1177/23779608211015154. PMC8114307 34017913

[51] M. M. Tse , S. S. Yeung , P. H. Lee , and S. S. Ng , “Effects of a Peer‐Led Pain Management Program for Nursing Home Residents With Chronic Pain: A Pilot Study,” Pain Medicine 17, no. 9 (2016): 1648–1657, 10.1093/pm/pnv121. 26893112

[52] H. van Gestel‐Timmermans , E. P. Brouwers , M. A. van Assen , and C. van Nieuwenhuizen , “Effects of a Peer‐Run Course on Recovery From Serious Mental Illness: A Randomized Controlled Trial,” Psychiatric Services 63, no. 1 (2012): 54–60, 10.1176/appi.ps.201000450. 22227760

[53] M. M. Y. Tse , E. Yan , A. S. K. Tang , D. Cheung , and S. Ng , “A Music‐With‐Movement Exercise Programme for Community‐Dwelling Older Adults Suffering From Chronic Pain: A Pilot Randomized Controlled Trial,” Nursing Open 10, no. 9 (2023): 6566–6574, 10.1002/nop2.1915. 37415289 PMC10416030

[54] J. E. S. Beauchamp , M. Wang , L. G. Leon Novelo , et al., “Feasibility and User‐Experience of a Virtual Environment for Social Connection and Education After Stroke: A Pilot Study,” Journal of Stroke and Cerebrovascular Diseases 33, no. 2 (2024): 107515, 10.1016/j.jstrokecerebrovasdis.2023.107515. 38064972

[55] R. Hardman , S. Begg , and E. Spelten , “What Impact Do Chronic Disease Self‐Management Support Interventions Have on Health Inequity Gaps Related to Socioeconomic Status: A Systematic Review,” BMC Health Services Research 20, no. 1 (2020): 150, 10.1186/s12913-020-5010-4. 32106889 PMC7045733

[56] P. Iovino , E. Vellone , N. Cedrone , and B. Riegel , “A Middle‐Range Theory of Social Isolation in Chronic Illness,” International Journal of Environmental Research and Public Health 20, no. 6 (2023): 4940, 10.3390/ijerph20064940. 36981849 PMC10049704

[57] E. Schoenmakers , M. Lasgaard , and J. Power , “Guidelines for Evaluating and Reporting Social Isolation and Loneliness Interventions,” Journal of Health Psychology 03/26 (2024): 338–352, 10.1177/13591053241238127. PMC11800726 38527950

[58] N. Morrish , S. Choudhury , and A. Medina‐Lara , “What Works in Interventions Targeting Loneliness: A Systematic Review of Intervention Characteristics,” BMC Public Health 23, no. 1 (2023): 2214, 10.1186/s12889-023-17097-2. 37946186 PMC10636966

[59] S. Byth , P. Frijters , and T. Beatton , “The Relationship Between Obesity and Self‐Esteem: Longitudinal Evidence From Australian Adults,” Oxford Open Economics 1 (2022): odac009, 10.1093/ooec/odac009.

[60] M. T. Jensen , S. S. Nielsen , C. Jessen‐Winge , et al., “The Effectiveness of Social‐Support‐Based Weight‐Loss Interventions—A Systematic Review and Meta‐Analysis,” International Journal of Obesity 48, no. 5 (2024): 599–611, 10.1038/s41366-024-01468-9. 38332127

[61] K. Ufholz , “Peer Support Groups for Weight Loss,” Current Cardiovascular Risk Reports 14, no. 10 (2020): 19, 10.1007/s12170-020-00654-4.

[62] M. Aguilera , “Post‐Surgery Support and the Long‐Term Success of Bariatric Surgery,” Practice Nursing 25, no. 9 (2014): 455–459, 10.12968/pnur.2014.25.9.455.

[63] E. Mamalaki , D. Poulimeneas , T. Tsiampalis , et al., “The Effectiveness of Technology‐Based Interventions for Weight Loss Maintenance: A Systematic Review of Randomized Controlled Trials With Meta‐Analysis,” Obesity Reviews 23, no. 9 (2022): e13483, 10.1111/obr.13483. 35686875

[64] B. Spring , J. M. Duncan , E. A. Janke , et al., “Integrating Technology Into Standard Weight Loss Treatment: A Randomized Controlled Trial,” JAMA Internal Medicine 173, no. 2 (2013): 105–111, 10.1001/jamainternmed.2013.1221. 23229890 PMC3684245

[65] R. C. Ambagtsheer , K. Borg , L. Townsin , et al., “The Effectiveness of Technology Interventions in Reducing Social Isolation and Loneliness Among Community‐Dwelling Older People: A Mixed Methods Systematic Review,” Archives of Gerontology and Geriatrics Plus 1, no. 1 (2024): 100008, 10.1016/j.aggp.2024.100008.

[66] C. Gardiner , G. Geldenhuys , and M. Gott , “Interventions to Reduce Social Isolation and Loneliness Among Older People: An Integrative Review,” Health & Social Care in the Community 26, no. 2 (2018): 147–157, 10.1111/hsc.12367. 27413007

