# Supplementary material for: Systematic Review of Loneliness and Social Isolation Interventions in Obesity and Obesity‐Related Complications
Source: Obes Rev. 2026 Jan 26;27(7):e70099. doi: 10.1111/obr.70099 (PMC13243346; doi:10.1111/obr.70099)
Supplement: Supplementary file 1 — Data S1: Supplementary Information. [file OBR-27-e70099-s001.pdf]

PRISMA

Supplementary Appendix 1

PRISMA 2020 Checklist

Section and Topic	Item #	Checklist item	Location where item is reported
**TITLE**			
Title	1	Identify the report as a systematic review.	Pg 1
**ABSTRACT**			
Abstract	2	See the PRISMA 2020 for Abstracts checklist.	Pg 2
**INTRODUCTION**			
Rationale	3	Describe the rationale for the review in the context of existing knowledge.	Pg 4
Objectives	4	Provide an explicit statement of the objective(s) or question(s) the review addresses.	Pg 4
**METHODS**			
Eligibility criteria	5	Specify the inclusion and exclusion criteria for the review and how studies were grouped for the syntheses.	Pg 5
Information sources	6	Specify all databases, registers, websites, organisations, reference lists and other sources searched or consulted to identify studies. Specify the date when each source was last searched or consulted.	Pg 5
Search strategy	7	Present the full search strategies for all databases, registers and websites, including any filters and limits used.	Supplementary A2
Selection process	8	Specify the methods used to decide whether a study met the inclusion criteria of the review, including how many reviewers screened each record and each report retrieved, whether they worked independently, and if applicable, details of automation tools used in the process.	Pg 5
Data collection process	9	Specify the methods used to collect data from reports, including how many reviewers collected data from each report, whether they worked independently, any processes for obtaining or confirming data from study investigators, and if applicable, details of automation tools used in the process.	Pg 5/6
Data items	10a	List and define all outcomes for which data were sought. Specify whether all results that were compatible with each outcome domain in each study were sought (e.g. for all measures, time points, analyses), and if not, the methods used to decide which results to collect.	Pg 5/6
	10b	List and define all other variables for which data were sought (e.g. participant and intervention characteristics, funding sources). Describe any assumptions made about any missing or unclear information.	Pg 5/6
Study risk of bias assessment	11	Specify the methods used to assess risk of bias in the included studies, including details of the tool(s) used, how many reviewers assessed each study and whether they worked independently, and if applicable, details of automation tools used in the process.	Pg 6
Effect measures	12	Specify for each outcome the effect measure(s) (e.g. risk ratio, mean difference) used in the synthesis or presentation of results.	NA
Synthesis methods	13a	Describe the processes used to decide which studies were eligible for each synthesis (e.g. tabulating the study intervention characteristics and comparing against the planned groups for each synthesis (item #5)).	Pg 6

PRISMA

Section and Topic	Item #	Checklist item	Location where item is reported
	13b	Describe any methods required to prepare the data for presentation or synthesis, such as handling of missing summary statistics, or data conversions.	NA
	13c	Describe any methods used to tabulate or visually display results of individual studies and syntheses.	Pg 6
	13d	Describe any methods used to synthesize results and provide a rationale for the choice(s). If meta-analysis was performed, describe the model(s), method(s) to identify the presence and extent of statistical heterogeneity, and software package(s) used.	NA
	13e	Describe any methods used to explore possible causes of heterogeneity among study results (e.g. subgroup analysis, meta-regression).	NA
	13f	Describe any sensitivity analyses conducted to assess robustness of the synthesized results.	NA
Reporting bias assessment	14	Describe any methods used to assess risk of bias due to missing results in a synthesis (arising from reporting biases).	NA
Certainty assessment	15	Describe any methods used to assess certainty (or confidence) in the body of evidence for an outcome.	NA
**RESULTS**			
Study selection	16a	Describe the results of the search and selection process, from the number of records identified in the search to the number of studies included in the review, ideally using a flow diagram.	Pg 5/6
	16b	Cite studies that might appear to meet the inclusion criteria, but which were excluded, and explain why they were excluded.	Pg 5
Study characteristics	17	Cite each included study and present its characteristics.	Pg 6-9
Risk of bias in studies	18	Present assessments of risk of bias for each included study.	Pg 9
Results of individual studies	19	For all outcomes, present, for each study: (a) summary statistics for each group (where appropriate) and (b) an effect estimate and its precision (e.g. confidence/credible interval), ideally using structured tables or plots.	NA
Results of syntheses	20a	For each synthesis, briefly summarise the characteristics and risk of bias among contributing studies.	NA
	20b	Present results of all statistical syntheses conducted. If meta-analysis was done, present for each the summary estimate and its precision (e.g. confidence/credible interval) and measures of statistical heterogeneity. If comparing groups, describe the direction of the effect.	NA
	20c	Present results of all investigations of possible causes of heterogeneity among study results.	NA
	20d	Present results of all sensitivity analyses conducted to assess the robustness of the synthesized results.	NA
Reporting biases	21	Present assessments of risk of bias due to missing results (arising from reporting biases) for each synthesis assessed.	NA
Certainty of evidence	22	Present assessments of certainty (or confidence) in the body of evidence for each outcome assessed.	NA

Section and Topic	Item #	Checklist item	Location where item is reported
**DISCUSSION**			
Discussion	23a	Provide a general interpretation of the results in the context of other evidence.	Pg 10
	23b	Discuss any limitations of the evidence included in the review.	Pg 12
	23c	Discuss any limitations of the review processes used.	Pg 12
	23d	Discuss implications of the results for practice, policy, and future research.	Pg 12
**OTHER INFORMATION**			
Registration and protocol	24a	Provide registration information for the review, including register name and registration number, or state that the review was not registered.	Pg 4
	24b	Indicate where the review protocol can be accessed, or state that a protocol was not prepared.	Pg 4
	24c	Describe and explain any amendments to information provided at registration or in the protocol.	NA
Support	25	Describe sources of financial or non-financial support for the review, and the role of the funders or sponsors in the review.	NA
Competing interests	26	Declare any competing interests of review authors.	Pg 1
Availability of data, code and other materials	27	Report which of the following are publicly available and where they can be found: template data collection forms; data extracted from included studies; data used for all analyses; analytic code; any other materials used in the review.	Pg 5

From: Page MJ, McKenzie JE, Bossuyt PM, Boutron I, Hoffmann TC, Mulrow CD, et al. The PRISMA 2020 statement: an updated guideline for reporting systematic reviews. BMJ 2021;372:n71. doi: 10.1136/bmj.n71. This work is licensed under CC BY 4.0. To view a copy of this license, visit https://creativecommons.org/licenses/by/4.0/

Supplementary Appendix 2

Full search strategy for each database searched.

Embase via Ovid

Search number	Search terms
1	chronic disease/ OR chronic*.tw. OR diabetes mellitus/ OR diabetes.tw. OR obesity/ OR obes*.tw. OR cardiovascular disease*.tw. OR hypertension.tw. OR heart failure*.tw. OR coronary artery disease*.tw. OR coronary heart disease*.tw. OR obstructive sleep apn?ea.tw. OR dyslipid?emia*.tw. OR metabolic syndrome*.tw. OR non?alcoholic fatty liver disease.tw. OR osteoarthritis.tw. OR mental health disorder*.tw. OR mental health condition*.tw. OR serious mental illness*.tw.
2	web-based intervention/ OR intervention.tw. OR intervention study/ OR psychosocial intervention/ OR trial.tw. OR clinical trial/ OR randomized controlled trial/ OR evaluation.tw. OR evaluation study/ OR pilot.tw. OR pre post.tw. OR controlled study/ OR program*.tw. OR model of care.tw. OR<br>service.tw. OR social prescribing.tw.
3	lone*.tw. OR loneliness/ OR social* isolat*.tw.
	de Jong Gierveld Loneliness Scale/ OR<br>de Jong Gierveld Loneliness Scale.mp. OR UCLA Loneliness Scale/ OR UCLA Loneliness Scale.mp. OR social disconnectedness scale.mp. OR Berkman Syme Social Network Index.mp. OR Steptoe Social Isolation Index.mp. OR duke social support index.mp. OR Lubben Social Network Scale.mp. OR Campaign to End Loneliness Measurement Tool.mp OR Revised UCLA Loneliness Scale/ Or scale*.mp. OR tool*.mp. OR measure*.mp. OR survey*.mp. OR index.mp.
5	1 AND 2 AND 3 AND 4

APA PsycINFO

Search	Search terms
1 AND	(TI chronic disease* OR AB chronic disease*) OR (TI chronic* OR AB chronic*) OR (TI diabetes OR AB diabetes) OR (TI obes* OR AB obes*) OR (TI cardiovascular disease* OR AB cardiovascular disease*) OR (TI hypertension OR AB hypertension) OR (TI heart failure* OR AB heart failure*) OR (TI coronary artery disease* OR AB coronary artery disease*) OR (TI coronary heart disease* OR AB coronary heart disease*) OR (TI dyslipid#emia OR AB dyslipid#emia) OR (TI metabolic syndrome OR AB metabolic syndrome) OR (TI obstructive sleep apn#ea OR AB obstructive sleep apn#ea) OR (TI osteoarthritis OR AB osteoarthritis) OR (TI non#alcoholic fatty liver disease* OR AB non#alcoholic fatty liver disease*) OR (TI mental health condition* OR AB mental health condition*) OR (TI serious mental illness* OR AB serious mental illness*) OR (TI psychiatric disorder* OR AB psychiatric disorder*)
2 AND	(TI web-based intervention OR AB web-based intervention) OR (TI intervention OR AB intervention) OR (TI intervention study OR AB intervention study) OR (TI psychosocial intervention OR AB psychosocial intervention) OR (TI trial OR AB trial) OR (TI clinical trial OR AB clinical trial) OR (TI randomized controlled trial OR AB randomized controlled trial) OR (TI evaluation OR AB evaluation) OR (TI evaluation study OR AB evaluation study) OR (TI pilot OR AB pilot) OR (TI pre post OR AB pre post) OR (TI controlled study OR AB controlled study) OR (TI program* OR AB program*) OR (TI model of care OR AB model of care) OR (TI service OR AB service) OR (TI social prescribing OR AB social prescribing)
3 AND	(TI lone* OR AB lone*) OR (TI loneliness OR AB loneliness) OR (TI social isolation OR AB social isolation) OR (TI social* isolat* OR AB social* isolat*)
4	(de Jong Gierveld Loneliness Scale) OR (UCLA Loneliness Scale) OR (social disconnectedness scale) OR (Berkman Syme Social Network Index) OR (Steptoe Social Isolation Index) OR (duke social support index) OR (Lubben Social Network Scale) OR (Campaign to End Loneliness Measurement Tool) OR (scale*) OR (survey*) OR (tool*) OR

	(index) OR (measure*)

SCOPUS

Search	Search terms
1	(de-jong-gierveld-loneliness-scale OR ucla-loneliness-scale OR social-disconnectedness-scale OR berkman-syme-social-network-index OR steptoe-social-isolation-index OR duke-social-support-index OR lubben-social-network-scale OR campaign-to-end-loneliness-measurement-tool OR survey* OR tool* OR scale* OR index OR measure*) AND TITLE-ABS-KEY (loneliness OR social-isolation OR lone* OR social*-isolat*) AND TITLE-ABS-KEY (chronic-disease* OR chronic* OR diabetes OR obes* OR cardiovascular-disease* OR hypertension OR coronary-heart-disease* OR coronary-artery-disease* OR heart-failure* OR dyslipid*emia OR non*-alcoholic-fatty-liver-disease* OR obstructive-sleep-apn*ea* OR osteoarthritis OR serious-mental-illness* OR mental-health-disorder* OR mental-health-condition* OR psychiatric-disorder*) AND TITLE-ABS-KEY (web-based-intervention OR intervention OR intervention-study OR psychosocial-intervention OR trial OR clinical-trial OR randomized-controlled-trial OR evaluation OR evaluation-study OR pilot OR pre-post OR controlled-study OR program* OR model-of-care OR service OR social-prescribing) AND NOT TITLE-ABS-KEY (child* OR cross-sectional OR case-control OR mouse OR mice OR animal) AND NOT TITLE (correlate* OR associate* OR cohort) AND (LIMIT-TO (DOCTYPE , "ar"))

Supplementary Appendix 3

Table of all data extracted from included studies

Study	Year	Country	Aim of study	Condition/s Studied	Study design	Total number of participants	% female	Age Overall	Outcome Measures	Description Intervention 1	Description Control	Length Intervention	Frequency	Group or individual	Setting	Intervention type	intervention components	Summary or loneliness/social isolation result	Summary of other outcomes	Limitations	Effective?
Adery 2022	2022	United States	To test the feasibility of conducting a choir group to examine the effects of a naturalistic and inclusive choral group intervention in schizophrenia-spectrum conditions (SCZ) and to explore and evaluate the effects of an 8-week choir intervention primarily targeting loneliness, and	SMI - schizophrenia-spectrum conditions (SCZ)	Pre post test	21	52 %	48.33 (10.4)	UCLA loneliness scale	1 hr choral rehearsal casual socialization (5 min) physical and vocal warm-ups in a circle (15 min) group learning and rehearsing of choral music (25 min)		8 weeks	weekly	Group	In person	Physical activity - singing	promote social interaction,	Matched-pairs analysis showed that participating in the choir significantly reduced loneliness (t[16] = 2.09, p = .021) with a mean difference score of 6.29 (pre M = 30.2, post M = 23.8; see Figure 1). Additionally, the number of sessions a participant attended was correlated with reduced loneliness (r = 4.15, p = .013).	Significant improvement at post-intervention was observed in the reduction of overall psychiatric symptoms as assessed by the BPRS (t[16] = 1.89, p = .032, mean difference = 2.17). However, changes in SAPS or SANS were not significant. Depression (BDI) scores were also significantly reduced at post-intervent	Small sample size lack of specificity of what is effective - due to the number of variables within the choir intervention itself, it is not possible to specify what factors contribute to observed benefits.	Yes

			symptoms.																ion (t[16] = 2.02, p = .026, mean difference = 2.05). No difference was found in self-perceived stress levels at post-intervention evaluation, as measured by the PSS. There was no significant change in CogState social emotional performance accuracy (pre = 81%, post = 84%)		
Ali 2021	2021	UK	The aim of this pilot trial was to assess the feasibility and acceptability of a future larger trial of	SMI - Depression	Randomised controlled trial	16	56 %	41.65 (15.2)	the Modified Worker Loneliness Questionnaire (MWLQ)	1 hour with matched friend volunteer with half the sessions in the community. Activities were recorded by the volunteer	access to the resource booklet in addition to usual care	6 months	once a week	individual	In person	Companionship - befriending	provide social support, social skills training	There were no statistically significant changes between baseline and 6 month follow up for befriendi	Depressive symptoms were lower in the intervention group compared with the control group at	issues with recruitment could not achieve 12 month follow up self-reported	No

			one-to-one befriending by volunteers in people with Intellectual disability (ID) and depressive symptoms							in a structured log-book. Monthly supervision was provided to volunteers by the volunteer coordinator and was recorded on a supervision form.								ng group participants for aloneness and social dissatisfaction, or between control and intervention group.	6 months [mean GDS-LD scores of 12.9 (SD: 6.7) and 17.5 (SD: 6.5), respectively]. After adjustment for depressive symptoms at baseline, the GDS-LD depressive symptoms score was four points lower in the intervention group compared to the control group (mean difference: 4.0; 95% confidence interval: 11.2 to 3.2). As expected for a pilot study, this was not statistically	measures	

																			significant. This change is equivalent to an effect size of 0.5 standard deviations		
Beauchamp 2024	2024	United States	To evaluate the feasibility and usability of stroke survivor participation in an 8-week virtual environment intervention that provides opportunities for social support exchanges, social network interactions, and recovery education	CVD - stroke	Pre post test	15	67 %	44	20 item UCLA loneliness scale	Continuous access to the Virtual Environment intervention, where two meetings or activities took place each week and support group meetings were held weekly, facilitated by a trained psychologist and/or licensed social worker/nurse, and guided by stroke support group meeting guidelines and education		8 weeks	Attendance in at least two meetings in the VE	Group	Technology - virtual environment	Therapeutic - support group for stroke	Promote social interaction, provide social support	non-significant changes	non-significant changes in social support, depression and anxiety, and self-efficacy	Results are limited by a small feasibility sample and lack of a control group. Selection bias may have occurred from use of convenience sampling	No
Berkman 2003	2003	United States	To determine whether mortalit	CVD - coronary heart disease	Randomised controlled trial	2481	44 %	61 (12.6)	ENRICHD Social Support Instrum	CBT (depression focused, social support	Usual care by physician	12 months The maximum	Either once a week, or more	both	In person	Therapeutic - therapy	promote social interaction,	For patients enrolled on the basis of	Four-year survival curves showed	6-month time frame may be insuffici	Yes

			y and recurrent infarction are reduced by treatment of depression and low perceived social support (LPSS) with cognitive behaviour therapy (CBT), supplemented with a selective serotonin reuptake inhibitor (SSRI) antidepressant when indicated, in patients enrolled within 28 days after MI.						ent (ESSI)	focused, or both) individually and group therapy after at least 3 individual sessions. patients with scores higher than 24 on the HRSD or those who showed a less than 50% reduction in BDI scores after 5 weeks were referred to study psychiatrists for consideration of pharmacotherapy. Unless contraindicated, sertraline hydrochloride (donated by Pfizer Inc, New York, NY, and provided without charge to intervention group patients, as needed) was initiated at 50 mg/d		duration of the behavioural intervention was 6 months. Group therapy could extend an additional 12 weeks and adjunctive pharmacotherapy for up to 12	if needed				social cognitive training, social skills training	LPSS, the mean ESSI score at 6 months and the mean increase in ESSI score from baseline were significantly higher in the intervention than the usual care group (24.4 vs 22.6 and 27% vs 18%, respectively). Between-group differences in ESSI scores diminished over time, primarily because of improvement in the usual care group. No benefit of the intervention on remained by 42 months	no significant difference between treatments in recurrence of MI or death (log-rank P = .94). This null effect was consistent for all secondary end points, including recurrent nonfatal MI, death from any cause, and cardiac death.	ent to address social support	

										and adjusted to a maximum of 200 mg/d if deemed necessary by the treating psychiatrist. Alternative medications (another SSRI or nortriptyline hydrochloride) were considered for patients unable to tolerate sertraline or judged unresponsive.								for the ESSI (P = .10).			
Forbes 2020	2020	Australia	To develop and pilot a 2 day intensive compassion focused therapy-based group program targeting weight stigma in women with overweight and obesity	Overweight and obesity	Pre post test	15	100 %	43.6 (12.38)	UCLA loneliness scale	CFT based, face-to-face, group intervention targeting weight stigma in females with overweight and obesity		two weeks	weekly (5 sessions per week)	group	In person	Therapeutic - therapy	promote social interaction, social cognitive training,	There was a significant improvement in loneliness from pre-intervention to post-intervention.	Significant increase in self-compassion with a large effect size. Pairwise comparisons revealed significant increases in self-compassion from pre- to post-intervention and from	the results presented here are preliminary. Significant improvements were found on many variables, yet the study comprised a small sample of Caucasian	Yes

																			pre-intervention to 3-month follow-up. Internalized weight stigma was found to decrease significantly over time with a large effect size. Pairwise comparisons indicated significant decreases in internalized weight stigma from pre- to post-intervention, and from pre-intervention to 3-month follow-up. Pairwise comparisons indicated significant	female participants who were self-selected. This therefore limits generalizability beyond the current study.	

																			improvements in psychological distress, life satisfaction, body dissatisfaction, body shame, and eating self-efficacy from pre-intervention to post-intervention, and from pre-intervention to 3-month follow-up. The results indicated a non-significant trend of mean group weight loss from pre-treatment to 3-month follow-up. Of the sample, eleven participants achieved		

																			weight loss from pre-intervention to 3-month follow-up, and the group achieved an average weight loss of 3.8 kg (8.37 pounds).		
Fortuna 2022	2022	United States	assess the feasibility, acceptability, and preliminary effectiveness of digital peer support integrated medical and psychiatric self-management intervention (PeerTECH) for adults with a serious mental illness.	SMI	Pre post test	21	71 %	39.85 (12.41)	20-item UCLA Loneliness Scale	PeerTECH is a mobile technology platform designed to facilitate the delivery of evidence-based principles that have been shown to promote self-management in people with SMI		12 weeks	4 times a month, 3 times a week text	individual	Technology - digital platform	Therapeutic	provide social support, social cognitive training, social skills training	Promising evidence of improvement (though not statistically significant) was demonstrated on positive changes in feelings of loneliness as measured by the UCLA loneliness measure.	On average, patient-participants demonstrated a significant increase in self-efficacy to manage the chronic disease as measured by the Self-Efficacy for Managing Chronic Disease Scale. They also demonstrated an average change	Pilot, so small sample size so not powered to detect statistically significant pre/ post differences no control group	No

																			on the Empowerment Scale from 2.12 at baseline to 1.77 post-treatment. Lower scores on the Empowerment Scale indicated higher levels of empowerment. Promising evidence of improvement (though not statistically significant) was demonstrated on positive changes in psychiatric self-management as measured by the IIMR scale, medical self-management as measured by the		

																			SHRAPS. Decreases were found related to negative changes in hope and social support.		
Ghahari 2015	2015	Australia	To compare the outcomes of the diabetes-specific self-management program (Diabetes) and the generic chronic disease Self-management Program (Chronic Condition) and to explore whether program characteristics provide insight into the results of the outcome evaluation.	Diabetes	Pre post test	290	53 %	64.1 (10.3)	Scale for social isolation Duke Loneliness Scale	People with diabetes self-selected attendance at the Diabetes or Chronic Condition program offered as part of routine practice.		6 weeks	weekly	group	in person	therapeutic (discussion and education)	promote social interaction	There were no statistically significant changes in loneliness and social isolation between groups or at different time points.	significantly more people in the Chronic Disease program (22.4% at post-test and 29.0% at follow up) made clinically important improvements in their self-efficacy than did those in the Diabetes program (17.6% at post-test and 15.1% at follow up).	self-reported no control group	No
Hoy-Gerlach 2022	2022	United States	Explore how ESAs	SMI	Pre post test	11	82 %	52.46	UCLA	each participant was		From the time of ESA		individual	in person	Companionship -	Provide social	there was a statistica	there was a statistic	Findings of this study	Yes

			may facilitate mental health recovery and related benefits for persons with Serious Mental Illness (SMI).					(7.51)		offered the option of adopting a dog (one year of age or old) or a cat or kitten		adoption to the completion of this study, each participant resided with their respective ESA about 12 months				animal companion	support	lly significant decreases in UCLA Loneliness Scale scores from Time 1 (M=59.20, SD=9.47) to Time 2 [M=49.90, SD=13.66, t(10)=3.80, p=.004]. The eta-squared statistic (.62) indicated a large effect size. Results further showed that 18 of 20 items on the loneliness scale scored a lower mean value after intervention; of these, 4 were statistically significant	ally significant decrease in Becks Depression Inventory (BDI) total scores from Time 1 (M=21.09, SD=8.43) to Time 2 [M=14.64, SD=7.03, t(10)=2.48, p=.03]. The eta-squared statistic (.41) indicated a large effect size. Results indicated that on the BDI scale 19 out of 21 items scored a lower mean value after intervention; 5 of which were statistically significant. there was a	are not generalizable; this was not a randomly selected or assigned sample, and there was no control or comparison group	

																			statistically significant decrease in Becks Anxiety Inventory (BAI) scores from Time 1 (M=23.55, SD=9.81) to Time 2 [M=17.73, SD=11.79, t(10)=2.24, p=.049]. The eta-squared statistic (.36) indicated a large effect size. There were not statistically significant changes in individual mean item scores on BAI.		
Koebner 2019	2019	United States	examine the feasibility of art museum tours (Art Rx) as an	Chronic pain	Pre post test	54	65 %	59 (14)	12-item social disconnection scale	Free, monthly docent-facilitated one-hour tours of the museum for any		One hour	11 tours, participants attended a single tour	group	in person	social activity	promote social interaction	Participants also had higher pre-intervention social disconne	The majority of participants (57%) stated that they	Its relatively small sample size and overall design (includi	Yes

			intervention for individuals with chronic pain.							individual with chronic pain and for their family members and/or friends. all participants were provided with lightweight stools to enhance comfort and facilitate accessibility. Tours took place in one gallery that was blocked off to the general public when possible, to facilitate a sense of connectedness and mitigate any potential mobility issues among participants. Tours included dialogue about 3-5 art objects selected ahead of time by the docents based on their								ction scores (M 26.00, SD 9.86) than postintervention (M 22.35, SD 9.86). This differenc 3.65 (BCa 95% CI 1.70-5.73) represented a moderate effect size (d 0.37).	experienced pain relief during the tour. Of those who experienced pain relief, the average pain relief reported (SD) was 47% (34.61%). On average, participants had higher pre intervention pain unpleasantness scores (M 4.02, SD 2.42) than postintervention (M 3.53, SD 2.61). This difference 0.49 (BCa 95% CI 0.06-0.90) represented a small effect size (d 0.20). Changes in pain intensity pre- to	ng the lack of a comparison group and randomization) make it susceptible to selection bias and do not allow for causal inferences, as alternative explanations of findings cannot be ruled out. In addition, chronic pain is a heterogeneous and complex condition with varied etiologies therefore, this study's broad self-reported inclusion criteria create another limitation to the	

										personal interest or expertise									post-tour were not significant, but they were significant at three-week follow-up (pre M 4.14, SD 2.24; three-week M 3.51, SD 2.48; difference 0.63, BCa 95% CI 0.07-1.25, P 0.034, d 0.28)	generalizability and interpretation of its results.	
Loi 2016	2016	Australia	Investigate whether an educational training course on using the internet and touchscreen technology (TT) would decrease social isolation and improve self-esteem in residents living	SMI	Pre post test	5	80 %	69.9	Hawthorne Friendship scale	Internet for seniors facilitated by the occupational therapist (OT) who worked in the unit and another staff member, usually a nurse.		6 weeks, 45 min	twice a week	individual	Technology - internet	Therapeutic - training	social cognitive training	No statistical differences	no statistical differences in self-esteem, friendship, or in the internet questionnaires.	very small sample size, no control	No

			in a low-level residential facility.																		
Muralidharan 2020	2020	United States	examine the effectiveness of in-person and online-delivered weight management interventions, compared to usual care, for improving health-related quality of life in adults with serious mental illness	SMI	Randomised controlled trial	276	6%	81.3 (9.4)	Three-Item Loneliness Scale	Online programming and peer coaching support. the same curriculum as in-person MOVE presented via text, audio, and video. Individuals could set goals and track their activity and weight. To facilitate engagement, peer coaches, who themselves were Veterans in recovery from serious mental illness, conducted weekly coaching calls with participants to provide reminders, support, and problem-solving. in-person weight manageme	Participants in usual care were given information on weight management, and could attend standard services, including the standard VA MOVE! program	6 months	30 modules, 24 sessions	Both	Both	Physical activity - exercise	provide social support, social cognitive training,	Comparing in-person MOVE and usual care, in-person MOVE was associated with a greater decrease in the Three Item Loneliness Scale total score at six months (t=âˆ’2.76, p=.006).	Comparing WebMOVE and usual care, there was a greater increase in IWQOL-SE at six months (t=2.23, p=.026). There were significant increases in both active interventions in VR-12 MCS compared to usual care: for WebMOVE, at three months (t=2.17, p=0.031) and six months (t=2.38, p=.018), and for in-person MOVE at six months (t=1.99,	participants were Veterans and mostly males; thus, findings may not generalize to other populations.	Yes

										nt program for Veterans who are overweight or obese. The in-person MOVE condition tested in the present study is a manualized version of MOVE!, tailored for adults with serious mental illness The sessions included psychoeducation, goal-setting, and weekly weigh-ins.									p=0.048). There were no significant group differences on any of the BASIS scales, General Life Satisfaction, IWQOL-PF, or VR-12 PCS.		
Saghaee 2020	2020	Other: Iran	The purpose of this study was to evaluate the effectiveness of Persian Diabetes Self-Management Education on self-efficacy, quality of life, self-care activity, depressi	Diabetes	Randomised controlled trial	34	41%	67.7 (7.1)	adult Social Emotional Loneliness Scale-Short form (SELSA-S)	PDSME - culturally appropriate diabetes education program	educational and non-interactive and conducted by the occupational therapist. At the end of each control class, participant's questions were answered by the	4 weeks	2 per week	group	In person	Therapeutic - education	promote social interaction	After the two-week follow-up, loneliness increased in both the intervention and control groups. However, after four weeks, the intervention group showed a decline in loneliness	a significant improvement in the medical self-efficacy domain, particularly for the intervention group as compared to the control group (p = 0.02).There was no	small sample size differences at baseline	No

			on and loneliness in older adults with type 2 diabetes.								educator.							s, indicating improvement in this variable, whereas the control group trend continued to increase. Ultimately, the results showed no significant change in the control and intervention groups in terms of loneliness after controlling for baseline data (p = 0.75).	significant difference between the intervention and controls group in the other domains of self-efficacy and questionnaire scores.<br><br>The overall DQOL score increased significantly more in the intervention group than the control group (mean difference between baseline and final score in intervention 4.41 ± 6.30 and control group 0.82 ± 5.27) (p Ë, 0.05). After the final		

																			follow-up, the participants of both groups showed an overall increase in the mean self-care scores compared to the baseline. The results showed no significant difference between the intervention and control groups (p = 0.71) Results showed that 82.7% of participants in the intervention group and 52.9% of participants in the control group had some degree of depressi		

																			on (mild, moderate, and severe). After four weeks of intervention, this percentage reached 64.6% and 58.8% in the intervention and control groups, respectively. Reported depression in the intervention group continued to improve over time, while worsening in the control group after two weeks before eventually decreasing after four weeks. Despite the PHQ-9 score decrease in the		

																			intervention group after the four-week duration, there were no significant differences between the control and intervention groups after the four-week follow-up (p = 0.38)		
Shih 2023	2023	Other: Taiwan	evaluate the effectiveness of AAT in improving social interactions and quality of life in patients with chronic schizophrenia during the COVID 19 pandemic.	SMI-schizophrenia	Randomised controlled trial	90	50 %	50.2 (9.6)	Mental health-social functioning scale (MHSFS)	support groups to help participants develop their skills in social interaction and emotional expression two service dogs and two professional AAT therapists. The dogs had received training for at least 3 months and were amicable. The AAT therapists	The control group participated in discussion groups once per week, sharing their thoughts about life; these activities were led by the researchers and social workers. For the	12 week 60 min	weekly	group	in person	Companionship - animal companion	promote social interaction, provide social support, social skills training	In the experimental group, the MHSFS scores of participants were significantly higher at T2 (M 52.80, SD 11.93) than at T1 (M 50.56, SD 11.89) (p < .01), but the MHSFS scores at T3 (M 46.07,	the experimental group, the SAFS scores of participants at T2 (M 9.87, SD 7.69) were significantly lower than those at T1 (M 11.56, SD 7.66) (p < .01). However, the decrease in SAFS scores at T3 (M 9.30, SD	small sample size	No

										had undergone at least 6 months of dog-related training and professional courses and had experience providing services to people with disabilities.	blinding of the participants, short films about animals were provided for the members of the control group to watch. Videos of cute animal documentaries were 10e15 minutes length each. Participants will then share their thoughts afterward. The intervention duration and frequency was identical for both groups							SD 14.36) were not significantly different from those at T1 ($p > .05$). Participants in the control group had significantly higher MHSFS scores at T2 (M 55.18, SD 14.34) than at T1 (M 54.09, SD 13.80) ($p < .05$), but their MHSFS scores at T3 (M 54.81, SD 12.97) were not significantly different from those at T1 ($p > .05$). The results revealed that both groups improved their social functioni	8.73) did not indicate a significant difference ($p > .05$). The control group had significantly lower SAFS scores at T2 (M 10.51, SD 8.21) and T3 (M 10.16, SD 7.46) than at T1 (M 11.87, SD 7.67) (both $p < .05$). The WHOQOL scores of the experimental group at T2 (M 86.42, SD 17.98) and T3 (M 86.64, SD 15.92) were significantly higher than their scores at		

																		ng after the intervention; however, this effect was not permanent.	T1 (M 79.33, SD 13.40) (both p < .01). The WHOQOL scores of the control group increased at T2 (M 81.02, SD 16.82) and decreased at T3 (M 75.16, SD 13.81); however, none of these differences were significant (all p > .05).		
Smith 2022	2022	United States	1) to describe and compare characteristics of participants and the CDSME work-shops they attended among those with complete	Chronic disease - sub analysis for diabetes and chronic pain	Pre-post	342	83%	74.28 (8.91)	The Campaign to End Loneliness Measurement Tool (CEL)	6 sessions in English or Spanish for diabetes and chronic pain (and general chronic disease though not included in extraction)		6 weeks	weekly	group	In person	therapeutic - education	promote social interaction	Significant differences were found between time points and loneliness scale scores. Loneliness scale scores improved from baseline to 6-weeks (3.16	NA	Self-reported, no comparison group, short time frame (6 weeks)	Yes

			loneliness data at both time points (i.e. at baseline and 6-weeks) compared to those who only had baseline loneliness data; (2) to identify participants with baseline scale scores indicative of loneliness based on participant and workshop characteristics; (3) to assess trends in loneliness scores from baseline to 6-weeks															±3.16 vs. 2.08±2.37, respectively) in the unadjusted model ($p < 0.001$) and the model adjusting for participant and workshop characteristics ($p < 0.001$). In the adjusted model, participants with improved loneliness scale scores from baseline to 6-weeks were younger ($\beta=-0.03$, $p=0.025$), Asian versus White race (mean difference =−1.57, $p < 0.001$), had a higher number of			

																		chronic conditions (β=0.17, p= 0.006), and attended the workshop at a faith based organization versus residential facility (mean difference =−1.09, p=0.035).			
Steinman 2021	2021	United States	evaluate PEARLS effectiveness for increasing social connectedness among underserved older adults with depression.	SMI-Depression	Pre post test	320	79%	72.6 (9.6)	Duke Social Support Index (DSSI-10) Patient-Reported Outcomes Measurement Information System Social Isolation (PROMIS-SI) 3 item UCLA-Loneliness	PEARLS providers meet one-on-one with participants during home visit. They help participants build problem-solving skills to gain a sense of control over overwhelming issues in their lives (Problem Solving Treatment [PST]), and plan meaningful and accessible physical,		4-6 months	8 times	individual	In person	Therapeutic - education	social cognitive training, social skills training	PEARLS participants were more socially connected at 6-month follow-up than at baseline. The mean(SD) change in social connectedness was 1.2(3.9) for DSSI-10, 2.2(6.2) for PROMIS-SI, and 0.5(2.2) for UCLA-Loneliness, with all three	Participants reduced their depression after PEARLS: the mean(SD) change in depression (PHQ-9) was 5.4(5.3) pre-post PEARLS	with no comparison group or randomization, we cannot attribute improvements in social connectedness to PEARLS specifically	Yes

										social, and pleasant activities (Behavioural Activation). Providers also offer psychoeducation (information and support to better understand and cope with depression) and linkages to social and health services when needed								scales showing statistically significant (p <0.001) changes at follow-up using paired t-tests (DSSI-10: t = 5.2, df = 312, p<0.001; PROMIS: t = 6.3, df = 310, p <0.001; UCLA: t = 3.7, df = 301,p <0.002). Sensitivity analysis of the DSSI-10 subscales using paired t-tests suggests most of the DSSI-10 change was driven by increased satisfaction with social support. Effect sizes were small to moderate for the DSSI-10			

																		(0.28), PROMIS-SI (0.35), and UCLA-Loneliness (0.21)			
Theeke 2021	2021	United States	Aim 1. Assess the feasibility and acceptability of implementing LISTEN in a sample of adult survivors of ischemic stroke who are experiencing loneliness.<br>Aim 2. Assess the initial efficacy of LISTEN for loneliness in a sample of adult survivors of ischemic stroke.	CVD - Stroke	Pre post test	6	50%	57 (6.23)	Loneliness (Revised UCLA Loneliness Scale)	LISTEN was originally designed to be delivered in group sessions to lonely people, aiming to help people with loneliness reframe problematic thoughts associated with loneliness. Participants of LISTEN attend five sequential 2 hour sessions; each beginning with writing (recording for those that cannot write), followed by exploration of five topics designed to facilitate the transition from thinking about loneliness		5 weeks, 2 hour sessions	one session a week	group	In person	Therapeutic - education/ discussion	promote social interaction, social cognitive training	Mean loneliness scores at enrolment were very high (mean 54.5, SD 5.08). In repeated measures comparisons of mean loneliness scores at baseline and 1,6, and 12 weeks post last LISTEN session, within subject loneliness scores did not change significantly [F (3, 15) 2.028, p .15, partial eta2 .289]. However, in paired t testing, mean loneliness scores	Mean depression scores indicated moderate depressive symptoms at enrolment with mean scores of 11.83 (SD 4.21). In repeated measures testing, mean depression scores did diminish to a score of 8.5 (SD 3.8) but this change was not significant. In paired t-tests, mean PHQ-9 scores diminished significantly from	The small sample size is a significant limitation to the study and diminishes the ability to generalize the study findings	Yes

										as insurmountable to a mindset of meaningfulness and solutions								from enrolment to 12 weeks post-LISTEN changed significantly (t 2.04, p .09). In addition, mean loneliness changed significantly (t 2.744, p .04) from1 week post last LISTEN session (mean 57.17, SD 6.24) to12 weeks post last LISTEN session (mean 51.83, SD 3.43) indicating participants continued to become less lonely	6-12 weeks post LISTEN (t 2.52, p .053). Participants improved on three subscales of the Neuro QoL. On applied cognition - executive function, participants reported less difficulty with executive function [F (3,15) 2.848, p .07, partial eta .363]. Participants reported enhanced positive affect and well-being [F (3,15) 3.156, p .056, partial eta .387] which indicates		

																			improvement on concepts such as hopefulness, purpose, meaning, balance, and life worth living. Finally, satisfaction with social roles steadily improved [F (3, 15) 3.889, p .031, partial eta .437] (see Figure 1). Repeated measures within subject analysis did not show significant decrease in systolic blood pressure [F (3, 15) 2.207, p .130, partial eta2 .306]. However, mean		

																			systolic blood pressure decreased from an average of 149 (SD 30.62) to a mean of 135 (SD 19.86) which is clinically relevant in stroke survivors		
Tse 2014	2014	Other: Hong Kong	Examine the feasibility of a peer-led pain management program among nursing home residents	Chronic pain	Randomised controlled trial	50	82 %	84.1 (9.2)	Chinese version of the UCLA Loneliness Scale	The experimental group (n=32) was given a 12-week group-based peer-led pain management program. There were two 1-hour sessions per week. Education in pain and demonstrations of nonpharmacological pain management strategies were provided. The research team and 12 trained peers led	The control group (n= 18) received one 1-hour session of pain management program each week over 12 weeks from the research team only.	12-week, 1 hour sessions	2 per week	group	In person	Therapeutic - education	provide social support, promote social interaction	Loneliness levels dropped significantly from 44.5 +_ 8.7 to 34.3 +_ 8.3 for the experimental group (p < 0.001) and from 42.8 6 10.5 to 38.9 +_ 9.8 for the control group (p = 0.031). There were no significant differences in outcome measures between the two	Both groups experienced a significant reduction in pain intensity from 5.8 +_ 2.6 at baseline to 3.4 +_ 2.5 after completing the intervention for the experimental group (p = 0.003) and from 6.3 +_ 3.0 to 3.1 +_ 2.4 for the control group (p = 0.001). ADL improve	self-reported data small sample size	No/Unclear

										the sessions. The control group (n ¼ 18) received one 1-hour session of pain management program each week over 12 weeks from the research team only								groups at both baseline and at week 12 (p > 0.05)	d significantly from 64.3 +_ 36.8 to 68.1 +_ 36.6 as measured by the MBI for the experimental group (p = 0.008) and from 65.6 +_ 30.9 to 71.1 +_ 31.5 for the control group (p = 0.014). In terms of psychological parameters, the happiness level grew from 16.8 +_ 5.3 to 20.6 +_ 4.4 post-intervention (P2) compared with the baseline (P1) for the experimental group (p < 0.001), while there		

																			was no statistically significant difference found in the control group.		
Tse 2023	2023	Other: Hong Kong	Test the effectiveness of a MMEP in mitigating pain intensity, pain self-efficacy and pain interferences, as well as reducing loneliness and depressive symptoms in a sample of community dwelling older adults in Hong Kong.	Chronic pain	Randomised controlled trial	71	72%	73.6 (9.5)	Chinese version of the 6-item De Jong Gierveld Loneliness Scale (DJGLS)	Each session lasted for 45 min, sessions consisted of warm-up and breathing exercises, strengthening and stretching exercises, music-with-movement exercises, and knowledge on pain and pain management Participants chose one to two songs from the five on the list and engaged in movement and exercise, then pain management education with a trained research assistant	usual care and a pain management pamphlet distributed by the healthcare professions. Usual care refers to the participant receiving their routine care.	8 weeks	twice a week	Group	In person	Physical activity	Promote social interaction,	No significant changes when compared to control	Significant reduction in pain intensity when compared to control, but not pain self-efficacy or depression	Limited sample size	No

van Gestel-Timmermans 2012	2012	Other: The Netherlands	Evaluate the effectiveness of a peer run course on the recovery of people with major psychiatric problems	Serious mental illness	Randomised controlled trial	333	66%	43.5 (10.5)	11 item de jong Loneliness Scale	Each group was led by two trained course instructors who had previously participated in the course themselves and who had then successfully Themes were the meaning of recovery to participants, personal experiences of recovery, personal desires for the future, making choices, goal setting, participation in society, roles in daily life, personal values, how to get social support, abilities and personal resources, and empowerment and assertiveness. The participants used a standardized		12 weekly two-hour sessions	weekly	group	In person	Therapeutic - education	promote social interaction, provide social support, social skills training	No effect was found on loneliness	The effect of the intervention (Cohens d) was small to moderate on empowerment and hope and small on self-efficacy beliefs, quality of life.	unclear precisely which ingredients contributed to the effect of the peer-run course and how often participants should attend the course to benefit from it	No

										workbook that described the theme of each session and contained assignments for the session and homework assignments completed a train-the trainer course											

Supplementary Appendix 4

The revised JBI critical appraisal tool for the assessment of risk of bias for randomised controlled trials. The last three questions excluded from the overall assessment as per the instructions in the tool.

Study ID	Ali 2021	Muralidharan 2020	Berkman 2003	VanGestel-Timmermans 2012	Tse 2014	Saghaee 2020	Tse 2024	Shih 2023
Was true randomization used for assignment of participants to treatment groups?	Yes	Unclear	Yes	Yes	Yes	Yes	Yes	Yes
Was allocation to treatment groups concealed?	Yes	No	No	Yes	Yes	Yes	Yes	No
Were treatment groups similar at the baseline?	No	Yes	Yes	Yes	Yes	No	Yes	Yes
Were participants blind to treatment assignment?	No	No	No	No	Yes	No	No	Yes
Were those delivering the treatment blind to treatment assignment?	No	No	No	No	No	No	No	No
Were outcome assessors blind to treatment assignment?	Yes	Yes	Unclear	Unclear	No	Yes	Yes	Unclear
Were treatment groups treated identically other than the intervention of interest?	Yes	Yes	Yes	Unclear	Yes	Yes	Unclear	Yes
Was follow up complete and if not, were differences between groups in terms of their follow up adequately described and analysed?	Yes	Yes	Yes	Yes	Yes	Yes	Yes	Yes
Were participants analysed in the groups to which they were randomized?	Yes	Yes	Yes	Yes	Yes	Yes	Yes	Yes
Were outcomes measured in the same way for treatment groups?	Yes	Yes	Yes	Yes	Yes	Yes	Yes	Yes
Were outcomes measured in a reliable way	Unclear	Yes	Unclear	Unclear	Unclear	Unclear	Yes	Unclear
Was appropriate statistical analysis used?	Yes	Yes	Yes	Yes	Yes	Yes	Yes	Yes
Was the trial design appropriate and any deviations from the standard RCT design (individual randomization, parallel groups) accounted for in the conduct and analysis of the trial?	Yes	Yes	Yes	Yes	Yes	Yes	Yes	Yes
% Yes	70%	60%	60%	60%	80%	70%	70%	70%

Supplementary Appendix 5

The NHLBI before-after (pre-post) studies with no control group assessment tool for included studies.

Study ID	Adery 2022	Ghahari 2015	Hoy-Gerlach 2022	Loi 2016	Koebner 2019	Theeke 2021	Forbes 2020	Steinman 2021	Fortuna 2022	Beauchamp 2024	Smith 2022
Was the study question or objective clearly stated?	Yes	Yes	No	Yes	Yes	Yes	Yes	Yes	Yes	Yes	Yes
Were eligibility/selection criteria for the study population prespecified and clearly described?	Yes	No	Yes	Yes	Yes	Yes	Yes	Yes	Yes	Yes	No
Were the participants in the study representative of those who would be eligible for the test/service/intervention in the general or clinical population of interest?	Yes	Yes	Yes	Yes	Yes	Yes	Yes	Yes	Yes	Yes	Yes
Were all eligible participants that met the prespecified entry criteria enrolled?	Cannot determine	Yes	Cannot determine	Cannot determine	Yes	No	No	No	Cannot determine	No	No
Was the sample size sufficiently large to provide confidence in the findings?	No	Cannot determine	Cannot determine	Cannot determine	Cannot determine	No	Cannot determine	Yes	Not reported	No	Yes
Was the test/service/intervention clearly described and delivered consistently across the study population?	Yes	Yes	Yes	Yes	Yes	Yes	Yes	Yes	Yes	Yes	Yes
Were the outcome measures prespecified, clearly defined, valid, reliable, and assessed consistently across all study participants?	Yes	Yes	Yes	Yes	Yes	Yes	Yes	Yes	Yes	Yes	Yes
Were the people assessing the outcomes blinded to the participants' exposures/interventions?	Cannot determine	No	No	Cannot determine	No	No	Not reported	No	No	Cannot determine	No
Was the loss to follow-up after baseline 20% or less? Were those lost to follow-up accounted for in the analysis?	Yes	No	Yes	Yes	Cannot determine	Yes	Yes	No	No	Yes	Yes
Did the statistical methods examine changes in outcome measures from before to after the intervention? Were statistical tests done that provided p values for the pre-to-post changes?	Yes	Yes	Yes	Yes	Yes	Yes	Yes	Yes	Yes	Yes	Yes
Were outcome measures of interest taken multiple times before the intervention and multiple times after the intervention (i.e., did they use an interrupted time-series design)?	No	No	Yes	No	No	Yes	No	No	No	No	No
If the intervention was conducted at a group level (e.g., a whole hospital, a community, etc.) did the statistical analysis take into account the use of individual-level data to determine effects at the group level?	Yes	Cannot determine	Cannot determine	No	Cannot determine	Cannot determine	Yes	No	No	No	Cannot determine

% Yes	50%	57%	58%	58%	58%	67%	67%	58%	50%	58%	58%
